# A clinical decision support tool for accurate hip fracture prediction: A nationwide cohort study

**DOI:** 10.1371/journal.pmed.1005190

**Published:** 2026-08-27

**Authors:** Kristian F. Axelsson, Henrik Litsne, Konstantinos Konstantinou, Hussnain Khalid, Aldina Pivodic, Mattias Lorentzon

**Affiliations:** 1 Sahlgrenska Osteoporosis Centre, Institute of Medicine, Sahlgrenska Academy, University of Gothenburg, Gothenburg, Sweden; 2 Region Västra Götaland, Närhälsan Norrmalm Health Centre, Skövde, Sweden; 3 APNC Sweden AB, Mölndal, Sweden; 4 Centre for Person-Centered Care (GPCC), Sahlgrenska Academy, University of Gothenburg, Gothenburg, Sweden; 5 Department of Medicine, Geriatrics and Emergency Medicine, Sahlgrenska University Hospital, Mölndal, Västra Götaland Region, Sweden; University of Exeter, UNITED KINGDOM OF GREAT BRITAIN AND NORTHERN IRELAND

## Abstract

**Background:**

Although hip fractures are commonly associated with functional decline, increased morbidity, and mortality, accurate models for both short- and long-term prediction that do not rely on in-person assessment remain lacking. The aim was to develop and validate a high-performing clinical decision support tool, that can be used for population screening without the need for patient assessment, for predicting hip fracture risk.

**Methods and findings:**

All individuals aged ≥50 years living in Sweden at baseline (randomly set between 2011 and 2013, *N* = 3,542,647), who had not been prescribed osteoporosis medication within the previous 2 years, were included and followed through the end of 2021. During follow-up, 142,327 individuals sustained a hip fracture. Using a broad unconditional approach, 139,980 variables encompassing diagnoses, medications, procedures, demographics and socioeconomic data with multiple historic windows and level of detail were defined. The dataset was divided into discovery (25%), development (65%) and holdout (10%) cohorts. The risk of hip fracture was evaluated using traditional Cox models, as well as machine learning methods XGBoost and DeepSurv. The developed clinical support tool FRACTURE-ML based on DeepSurv using 2,500 predictors yielded an area under the curve (AUC) of 0.89 (95% CI 0.88, 0.89) at year 1, 0.88 (95% CI 0.87,0.88) at two years, and a reduced model with 35 predictors yielded similar AUCs, 0.87 at 2 years and 0.85 at 5 years. Traditional Cox models with 35 and 400 predictors reached similar AUCs. Both the DeepSurv and the Cox models performed excellently at the individual level based on calibration plot analysis. Screening using the in Sweden advocated fracture liaison services (FLS) secondary prevention approach (recent fracture), resulted in an AUC of 0.55 (95% CI 0.54, 0.55) at two years. For 2-year prediction, FRACTURE-ML, which could be used as a complementary approach for primary prevention, identified nearly seven times more persons at risk (sensitivity 0.84 (95% CI 0.82, 0.85) versus 0.12 (95% CI 0.11, 0.13) than the FLS approach, with limited reduction in specificity (0.79 (95% CI 0.79, 0.79) versus 0.98 (95% CI 0.98, 0.98), respectively). The lack of external validation and implementation studies represents a limitation, as such studies are needed to establish the clinical usefulness of FRACTURE-ML.

**Conclusions:**

FRACTURE-ML was effective in predicting hip fracture and could be used as a resource-efficient solution for population screening to improve the prevention of hip fracture.

## Author summary

**Why was this study done?:**

- Hip fractures are common in older adults and often lead to serious outcomes such as disability, illness, and increased risk of death.
- Existing tools to predict fracture risk usually require patient-provided information, making large-scale screening difficult.
- There is a need for an accurate and simple way to identify people at high risk without requiring direct patient assessment.

**What did the researchers do and find?:**

- The model identified about seven times more high-risk individuals than current clinical practice, while maintaining relatively high precision.
- They developed a prediction model (FRACTURE-ML) using routinely collected health data, which showed high discriminative performance (AUC of up to 0.89) and excellent calibration.
- The researchers analyzed data from over 3.5 million individuals aged 50 years and older in Sweden and followed them for up to 10 years.

**What do these findings mean?:**

- The lack of external validation and implementation studies represents a limitation, as such studies are needed to establish the clinical usefulness of FRACTURE-ML.
- This approach could help target preventive measures more efficiently and potentially reduce the number of hip fractures.
- The findings show that it is possible to predict hip fracture risk at the population level without direct patient interaction.

## Abstract 3

In this nationwide cohort study, Kristian F. Axelsson and colleagues develop and validate clinical decision support tool for predicting hip fracture risk that can be used for population screening without the need for patient assessment.

## Introduction

Hip fractures are associated with significant morbidity, disability, mortality and substantial healthcare costs [1–4]. Therefore, it is of utmost importance to identify individuals at high risk of hip fracture in order to initiate effective preventive measures, such as treatment with osteoporosis medications and fall prevention [5–8]. As recently reviewed in JAMA, the US Preventive Services Task Force concluded that screening for osteoporosis to prevent osteoporotic fractures in postmenopausal women has moderate net benefit [9].

Several risk tools to evaluate the risk of hip fracture are available and include FRAX, QFracture and CFracture, but these algorithms are dependent on patient interaction due to their implementation of body mass index (BMI) and lifestyle information on smoking and alcohol in the algorithms [9–12]. A risk model sourced on Danish registry data and developed using traditional statistical methods had high accuracy, but did not consider competing risk of death [13,14]. Accurate risk algorithms based on electronic health records alone would allow cost-efficient population screening and an opportunity for prevention without a large impact on the healthcare system.

Patients sustaining a first fracture have an increased risk of recurrent fracture, especially during the first 2 years following the index fracture [15–17]. Therefore, structured secondary prevention programs known as Fracture Liaison Services (FLSs) targeting patients with recent fractures have been recommended and launched worldwide [18,19], resulting in higher rates of bone mineral density (BMD) testing, treatment initiation, improved medication adherence, and a decrease in the risk of recurrent fracture [20,21]. The Swedish National Board of Health and Welfare has identified the establishment of FLSs as a top national priority [22]. Although implementation is strongly advocated in most Western countries, and known to be important for those with osteoporotic fracture, general primary screening for high fracture risk is not.

We hypothesized that machine learning algorithms and traditional statistical models applied to national electronic health record data from more than 3.5 million individuals aged 50 years or older could be used to develop and validate *Fracture Risk Assessment and Classification Using Real-world Evidence and Machine Learning* (FRACTURE-ML), a clinical decision support tool capable of accurately predicting both short- and long-term hip fracture risk.

## Methods

### Study design

**Fig 1:**
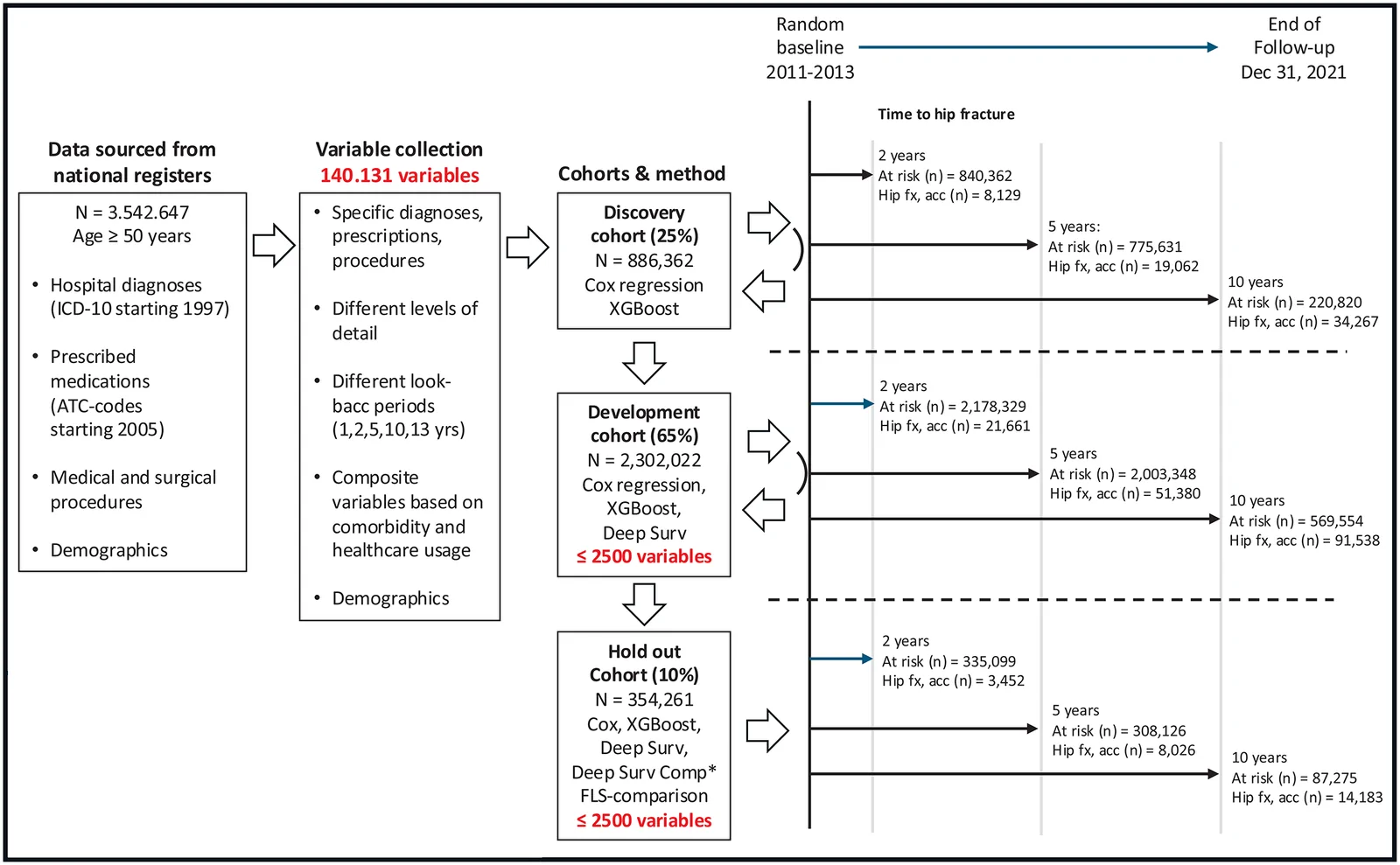
Study overview. Brief overview including data sources, variables, method workflow and follow-up. Hip fx acc (*n*) denotes the number of hip fractures accumulated after 2, 5 and 10 years follow-up. *DeepSurv Comp is a DeepSurv model not censored for death corresponding to a competing risk model.

This nationwide cohort study used multiple national registers in Sweden to develop a clinical decision support tool for hip fracture. A dataset of all Swedish men and women born 1981 or earlier and alive in 2005 was used. All individuals were assigned a random baseline date between 2011 and 2013. Only those aged 50 years or older and alive at baseline were included in the study population. Furthermore, individuals with recent (≤2 years) osteoporosis medication were excluded, rendering a population size of 3,542,647 individuals. A broad and unconditional approach was used to identify and create 139,980 baseline variables and possible predictors (Fig 1 and extended methods in S1 Appendix). Incident hip fracture was defined as fractures of the femoral head, neck, trochanter or subtrochanteric part of the femur accompanied with a code for surgical procedure and censored for death, emigration and end of study (December 31st, 2021). The whole dataset was divided into three separate cohorts for (i) discovery (25%), (ii) development (65%) and (iii) holdout (10%). The risk of hip fracture was evaluated yearly up to 10 years using three different methods: Cox regression, deep learning generalization of Cox regression (DeepSurv) with and without considering competing risks, and XGBoost for survival analysis. Ethical approval was obtained from the Swedish Ethical Review Authority (2022-00796-01). The requirement for informed consent was waived due to the use of anonymized participant data. This study is reported as per the Transparent Reporting of a multivariable prediction model for Individual Prognosis Or Diagnosis using machine learning or Artificial Intelligence (TRIPOD+AI) guideline (S1 Checklist).

### Data sources

Information regarding fractures and comorbidities was retrieved from The National Patient Register which includes hospital-based diagnoses from both inpatient and outpatient visits and uses ICD-10 since 1997. Socioeconomic data were retrieved from the LISA Register (Statistics Sweden), and date of death from Swedish Cause of Death Register (The National Board of Health and Welfare). Medication data was collected from The Swedish Prescribed Drug Register, starting July 1st, 2005. In Sweden, every resident receives a personal ID number at birth or immigration, allowing linkage across registries.

### Data partitioning and sample size justification

The total cohort was divided into discovery, development, and holdout sets which is essential for building reliable and generalizable models. The discovery set was used to explore patterns and to generate hypotheses, while the development set allowed for model training and tuning without biasing results. The holdout set, kept separate, provided an unbiased evaluation of how the model performed on new, unseen data. This separation helped to prevent overfitting, ensured that the findings were not artifacts of a specific dataset, and increased the confidence that the results will replicate in real-world applications. Sample size calculations for the development and holdout cohorts were performed using the three criteria of Riley and colleagues [23,24]. As a result, the development cohort consisted of 2,302,022 participants (65% of study population), the holdout cohort of 354,261 participants (10% of study population) and the discovery cohort of 886,362 participants (25% of study population) (for extended methods, see S1 Appendix).

### The discovery phase

Starting with 139,980 variables, age and sex adjusted univariable Cox models were fitted to eliminate insignificant predictors. The Benjamini–Hochberg [25] stepdown procedure was used as a multiple testing adjustment and variables with large standard errors were filtered out. Remaining candidate variables were evaluated using both multivariable Lasso Cox, and XGBoost in clinically adjacent groups, and gradually merged with other significant variables (S1 Fig). The 2500 most important variables were selected for model development.

### The model development phase

Subsampling techniques were used to deal with data imbalance and optimize the predictive performance of the models (DeepSurv, XGBoost, Cox). The tradeoff between predictive performance and model complexity was also investigated. The models were adequately tuned. The following models were evaluated: DeepSurv (2500 and 35 variables), DeepSurv competing (35 variables) [26], XGBoost (2500 and 35 variables) and Cox (400 and 35 variables). To avoid overfitting of the model, the loss from the internal validation set, a dataset from the development cohort used for model hyperparameter optimization was tracked, i.e., the partial likelihood, for each epoch, and stopped training when the validation loss started to increase (S2 Fig). To correct for an observed consistent overestimation of risk, the predicted probabilities from the DeepSurv and Cox models were recalibrated changing the intercept and slope of the calibration curves to minimize the sum of the squared errors (S3 Fig). The XGBoost model did not allow for individual probability calculations and could therefore not be calibrated.

### Validation in the holdout cohort

For prediction, the hip fracture probabilities from the discovery cohort were used to select Youden cutoffs. The models were evaluated for different time periods in the holdout cohort, and results are presented with receiver-operating characteristic curves (ROC), area under the curve (AUC), sensitivity, specificity, positive (PPV) and negative predictive values (NPV), calibration plots, and confusion matrix parameters. Finally, the predictive power of risk factors was investigated using permutation importance with change in AUC as statistic, i.e., variable importance was defined as how much AUC would have changed if the variable had no predictive value.

### Clinical implications

A comparison of the best models to FLS represented by any fracture the past year, was made. Furthermore, to quantify the clinical usefulness of the developed models, net benefit was calculated for different thresholds and models, and then compared to screen all, screen none and screening using FLS. To further demonstrate the clinical implications, hip fracture risk within 2 years for selected health profiles and age groups, as predicted by the model for women and men were presented. Two scenarios—one prioritizing high sensitivity (80%) and the other high specificity (80%)—were presented to illustrate effects of algorithm implementation.

### Statistical analyses

Descriptive baseline statistics for the most common diagnoses, medications, medical and surgical procedures, socioeconomical and previous fracture history variables are presented in terms of counts with percentage for categorical variables and averages with standard deviations (SD) for continuous variables. Statistical analyses were performed using Python version 3.11 using libraries such as Pycox, Scikit-Learn, and XGBoost, and R software version 4.4 for sample size calculation and some visualizations. Computations were performed using distributed data processing and distributed training across multiple compute nodes in a high-performance environment (Bianca) hosted by Uppsala Multidisciplinary Centre for Advanced Computational Science (UPPMAX) and National Academic Infrastructure (NAISS) for sensitive personal data (Extended methods in S1 Appendix). No large language models have been used for the creation of this manuscript.

## Results

### Study population

A total of 3,542,647 persons were included in the study. During a median (interquartile range) follow-up of 9.04 (8.16, 9.98) years there were a total of 142,327 hip fractures and 754,051 (21.3%) deaths. Patients with incident hip fractures were generally older, more frequently women, had more prescribed medications, and greater comorbidity than those without (Tables 1 and S1). The discovery, development and holdout cohorts were well balanced in terms of baseline characteristics (S2 Table).

**Table 1:** Baseline cohort characteristics.

	All	Incident hip fracture (max. follow-up)		SMD
		Yes	No	
	*N* = 3,542,647	*N* = 142,327	*N* = 3,400,320	
Age, years—mean (SD)	65.9 (11.0)	77.5 (9.9)	65.4 (10.8)	1.13
Female sex—no. (%)	1,805,997 (51.0)	91,458 (64.3)	1,714,539 (50.4)	0.28
Urban residence—no. (%)	815,971 (23.0)	32,448 (22.8)	783,523 (23.0)	0.01
Sickness benefits*—no. (%)	208,633 (5.9)	2,929 (2.1)	205,704 (6.0)	−0.17
Education*, <9 years of schooling—no. (%)	660,938 (18.7)	53,689 (37.7)	607,249 (17.9)	0.51
Marital status*—no. (%)				
Unmarried	535,448 (15.1)	14,935 (10.5)	520,513 (15.3)	−0.13
Divorced	621,262 (17.5)	22,863 (16.1)	598,399 (17.6)	−0.04
Widow(er)	417,372 (11.8)	46,366 (32.6)	371,006 (10.9)	0.68
Married	1,858,377 (52.5)	56,286 (39.5)	1,802,091 (53.0)	−0.27
Citizenship at birth—no. (%)				
Swedish	2,985,555 (84.3)	128,212 (90.1)	2,857,343 (84.0)	0.17
Nordic	183,304 (5.2)	7,145 (5.0)	176,159 (5.2)	−0.01
European	204,704 (5.8)	4,973 (3.5)	199,731 (5.9)	−0.10
Asian	107,046 (3.0)	1,199 (0.8)	105,847 (3.1)	−0.13
Other	62,038 (1.8)	798 (0.6)	61,240 (1.8)	−0.09
Previous event—no. (%)				
Fracture, past year	85,845 (2.4)	9,113 (6.4)	76,732 (2.3)	0.27
Fracture, past 10 years	476,366 (13.4)	38,076 (26.8)	438,290 (12.9)	0.41
Fall injury, past year	56,833 (1.6)	4,313 (3.0)	52,520 (1.5)	0.12
Fall injury, past 10 years	333,111 (9.4)	19,413 (13.6)	313,698 (9.2)	0.15
Osteoporosis medications, past 2–5 years—no. (%)	55,229 (1.6%)	3,939 (2.8%)	51,290 (1.5%)	0.09
Systemic estrogens, past 5 years—no. (%)	128,780 (3.6%)	6,975 (4.9%)	121,805 (3.6%)	0.07
Charlson comorbidity index—no. (%)				
0	2,377,896 (67.1)	66,366 (46.6)	2,311,530 (68.0)	−0.46
1	416,840 (11.8)	24,822 (17.4)	392,018 (11.5)	0.18
2	426,323 (12.0)	25,563 (18.0)	400,760 (11.8)	0.19
3	153,047 (4.3)	12,361 (8.7)	140,686 (4.1)	0.22
≥4	168,541 (4.8)	13,215 (9.3)	155,326 (4.6)	0.22
Hospital admissions, past 5 years—no. (%)				
0	2,280,174 (64.4)	65,264 (45.9)	2,214,910 (65.1)	−0.40
1	589,901 (16.7)	29,138 (20.5)	560,763 (16.5)	0.11
2	266,806 (7.5)	17,140 (12.0)	249,666 (7.3)	0.18
≥3	405,766 (11.5)	30,785 (21.6)	374,981 (11.0)	0.33
Different prescriptions, past 5 years—no. (%)				
0	248,741 (7.0)	4,085 (2.9)	244,656 (7.2)	−0.17
1–6	1,098,688 (31.0)	24,868 (17.5)	1,073,820 (31.6)	−0.31
7–13	1,038,056 (29.3)	41,615 (29.2)	996,441 (29.3)	0.00
14 or more	1,157,162 (32.7)	71,759 (50.4)	1,085,403 (31.9)	0.40

Selection of variables from the 139,980 list of candidates split per incident hip fracture. * = missing values treated as separate categories (marital status 3.1%, sickness benefits 3.1% and education level 4.2%). See S1 Table for the 10 most common diagnoses, medications, medical and surgical procedures.

### Model performance

**Fig 2:**
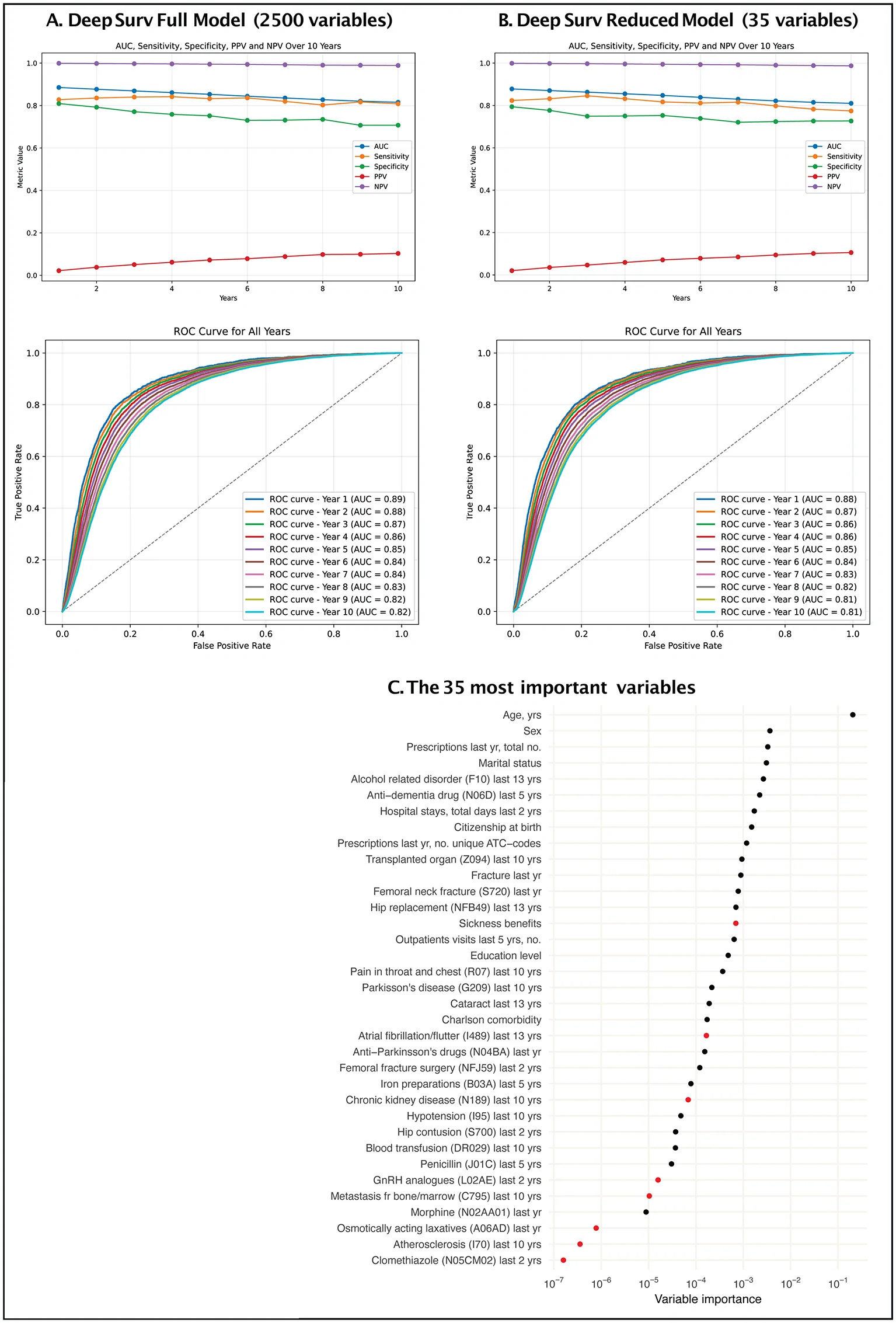
Performance of hip fracture prediction models. Deep Learning model (DeepSurv) performance in holdout cohort (*n* = 354,261) for **(A)** the full model using 2,500 variables and **(B)** a reduced model with 35 variables. Key confusion matrix parameters and ROC curves presented graphically for different follow-up periods. The most important variables in the DeepSurv 35 prediction model are presented with variable importance **(C)**, representing change in AUC when randomly permutated, i.e., how much AUC would have changed if the variable had no predictive value. Red dot denotes negative association. See S1 Appendix Extended Methods for variable details and characteristics.

All tested models were able to efficiently discriminate persons with and without incident hip fracture. The full DeepSurv model using 2500 variables resulted in an AUC of 0.89 (95% CI 0.88, 0.89) at year 1 (Fig 2A), which gradually declined with longer follow-up time to 0.88 (95% CI 0.87, 0.88) at year 2, 0.85 (95% CI 0.85, 0.86) at year 5 and 0.82 (95% CI 0.81, 0.82) at year 10. The sensitivity and specificity at year 1 was 0.83 (95% CI 0.81, 0.85) and 0.81 (95% CI 0.81, 0.81), respectively, and remained high year 2, 0.84 (95% CI 0.82, 0.85) and 0.79 (95% CI 0.79, 0.79), year 5 0.83 (95% CI 0.83, 0.84) and 0.75 (95% CI 0.75, 0.75)), and year 10 0.81 (95% CI 0.81, 0.81) and 0.71 (95% CI 0.71, 0.71) (S3 Table), respectively. The DeepSurv model with 35 variables performed similarly well with slightly lower 1-year AUC of 0.88 (95% CI 0.87, 0.89) (Fig 2B).

The XGBoost model performed similarly well and yielded an AUC of 0.89 (95% CI 0.88, 0.89) using the 2500 variable model at year 1 and resulted in slightly lower AUC values in the reduced model containing 35 variables. The Cox regression prediction results were nearly equivalent reaching an AUC of 0.87 (95% CI 0.87, 0.88) at year 1. Performance metrics for all models are presented in detail in S3 Table.

### Model parameters

In the reduced DeepSurv model with 35 variables, the key variables listed in the order of importance were age, sex, number of prescriptions the past year, marital status and alcohol related psychiatric disorder, with all 35 parameters listed in Fig 2C and variable characteristics provided in S4 Table.

### Calibration

**Fig 3:**
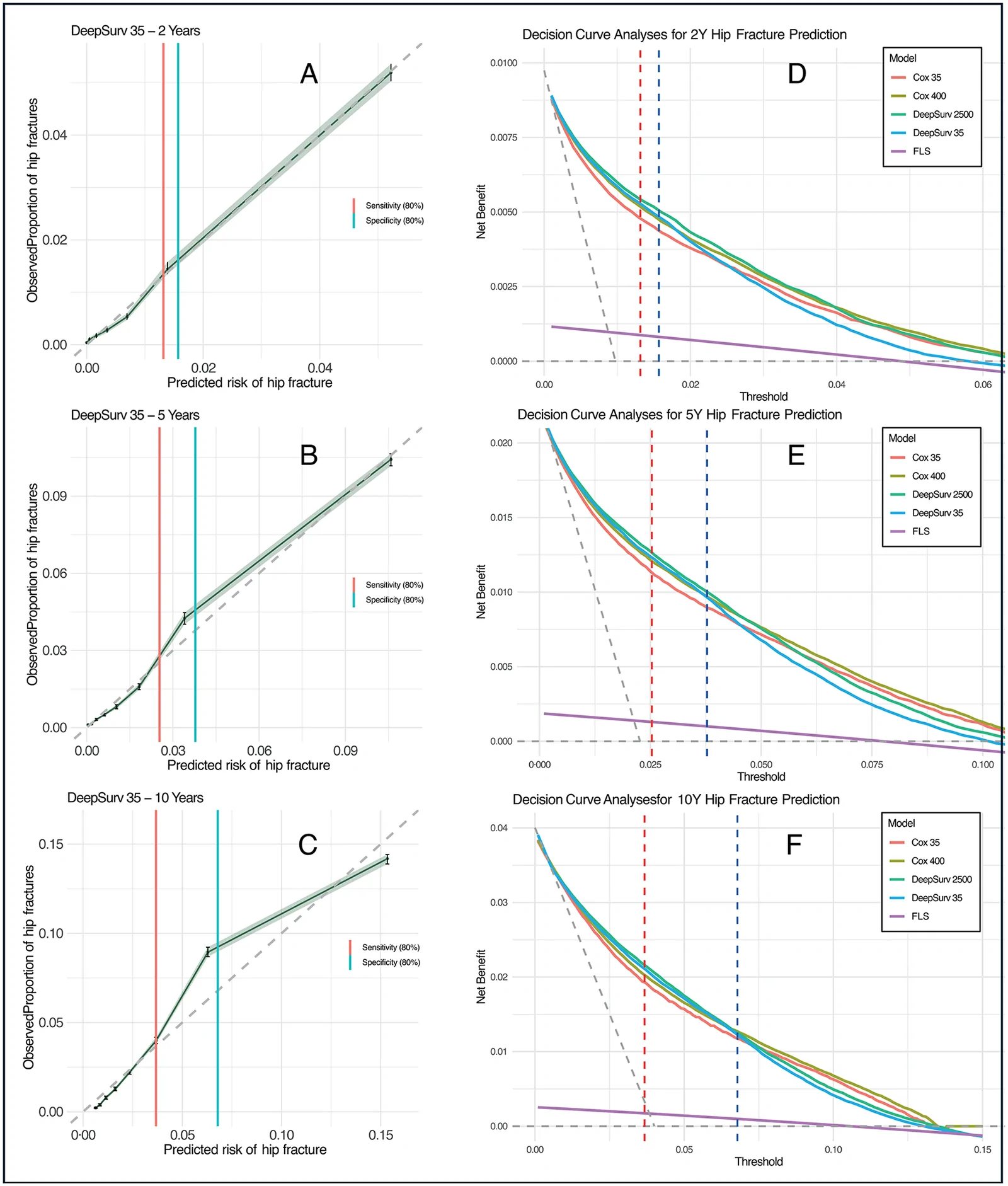
Calibration plots (A–C) and net benefit curves (D–F) in holdout cohort. Calibration plots for DeepSurv 35 were used to compare the predicted probabilities of hip fracture at 2 (A), 5 (B) and 10 (C) years with the observed events in the holdout cohort (*N* = 354,261). Thresholds for high-sensitivity (80%) and high-specificity (80%) are marked with red and green, respectively. For calibration plots with other methods, number of variables and follow-up time, see S4 Fig. Net benefit plots for hip fracture probabilities at 2 (D), 5 (E), and 10 (F) years for DeepSurv 35, DeepSurv 2,500, Cox 35, Cox 400 and FLS. Net benefit was calculated as (TP − FP × PB/(1 − PB))/*N* where TP = true positives, FP = false positives, PB = threshold probability, N = observations using the holdout cohort (*N* = 351,261). Default strategies screen all and screen none are represented as dark gray-dotted lines. Threshold probabilities representing 80% sensitivity (red) and 80% specificity (blue) are marked for DeepSurv 35.

Calibration plots for DeepSurv 2500, DeepSurv 35, Cox 35 and Cox 400 were used to compare the predicted probabilities of hip fracture at 2, 5 and 10 years against the observed events, to determine model performance on an individual level. Both Cox models (35 and 400) and the DeepSurv (2500 and 35) models generated predicted probabilities highly aligned with observed probabilities regardless of risk level, especially 2 years using the DeepSurv 35 model (Figs 3A–3C and S4).

### Mortality and competing risk

In a DeepSurv model with the 35 most important variables not censoring for death to assess the impact of competing risk of death, the model still performed well with AUC after 1, 2, 5 and 10 years of 0.88 (95% CI 0.87, 0.89); 0.87 (95% CI 0.87, 0.88); 0.85 (95% CI 0.85, 0.85) and 0.81 (95% CI 0.81, 0.81), respectively. All confusion matrices and performance parameters are presented in S3 Table.

### Comparison with the fracture liaison services approach

To assess the viability of the DeepSurv35 model compared to the FLS approach (based on the presence of a fracture in the past year and frequently used in clinical practice), the hip fracture probability at 2, 5 and 10 years were compared (S5 Fig). The FLS model resulted in an AUC of 0.55 (95% CI 0.54, 0.55) at two years (S3 Table). In comparison, the DeepSurv35 model at 2 years identified nearly 7 times as many patients at risk (sensitivity 0.83 (95% CI 0.82, 0.84) versus 0.12 (95% CI 0.11, 0.13) respectively, with both models having high specificity (0.78 (95% CI 0.78, 0.78) vs. 0.98 (95% CI 0.98, 0.98)) (S5 Fig and S3 Table).

### Decision curve analyses

The net benefit, i.e., the trade-off between true positives and false positives, for different threshold probabilities to show that the model is better than screening all or none, is presented for DeepSurv 2500, DeepSurv 35, Cox 400, Cox 35 and compared to the FLS approach. All models showed added net benefit compared to FLS and screen all/none within the relevant threshold intervals. (Fig 3D–3F).

### Clinical implications

**Fig 4:**
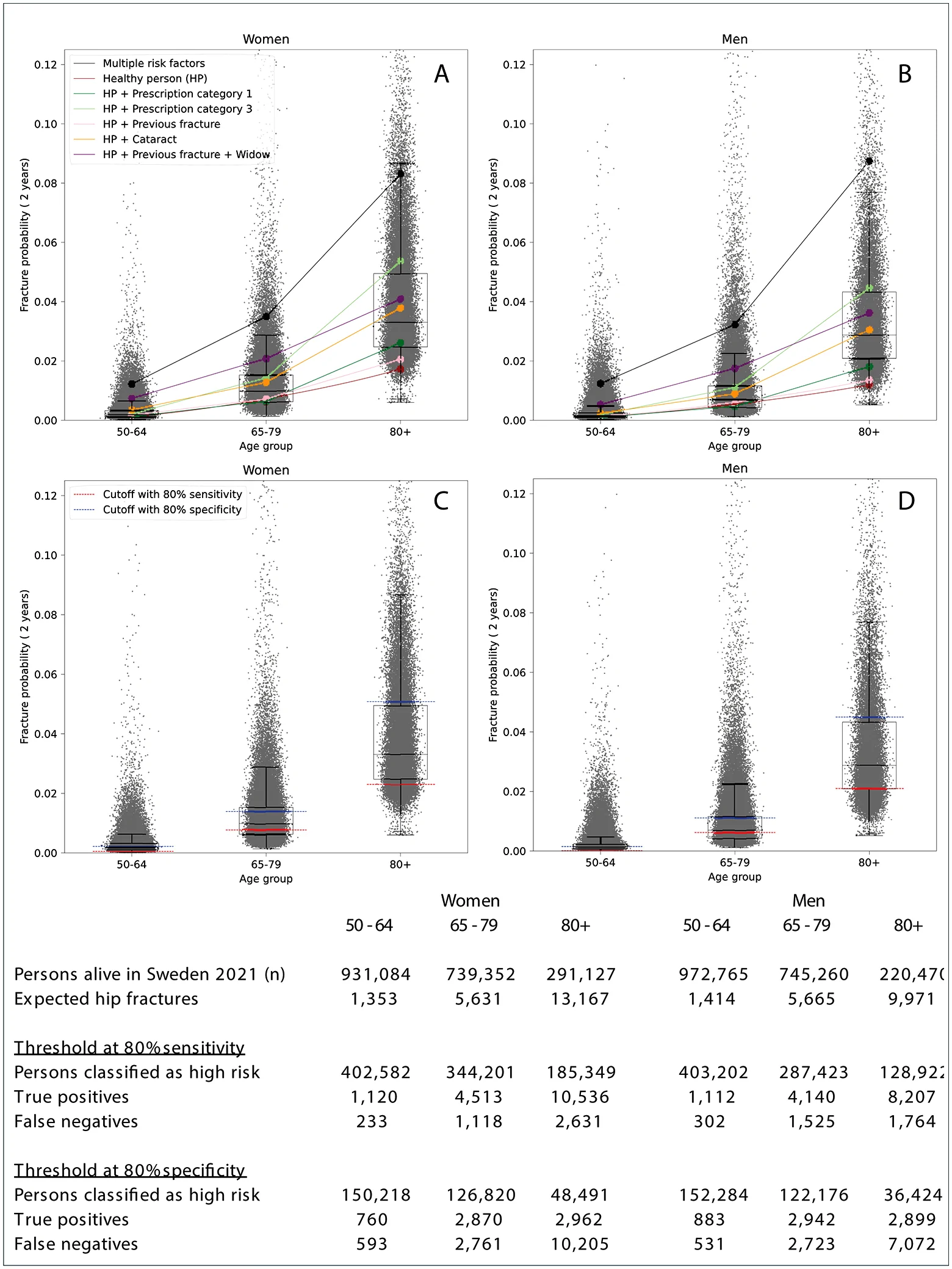
Examples of clinical cases and clinical implications. Hip fracture risk within 2 years for selected health profiles and age groups, as predicted by the model (DeepSurv 35) for women **(A)** and men **(B)**. Visualization of thresholds at 80% sensitivity and 80% specificity for women **(C)** and men **(D)** with corresponding numbers identified.

To illustrate clinical implications and predicted probabilities of the model for selected health profiles, ranging from a healthy person to those with multiple risk factors, the probabilities for hip fracture risk within 2 years are presented for men and women per age groups (Fig 4A and 4B). Probability thresholds dependent on either high (80%) sensitivity or high (80%) specificity were used. For example, using an 80% sensitivity probability threshold on 739,352 women 65−79 years old (the total population), 344,201 would be classified as high risk, of which 4,513 would suffer a hip fracture (true positives), whilst 1,118 patients would sustain a hip fracture but have probabilities below the threshold (false negatives). In comparison, using an 80% specificity threshold probability, would identify 126,820 women of which 2,870 true positives and 2,761 false negatives (Fig 4). An alternative illustration of clinical implication using precision-recall curves is presented with predictions for 2, 5 and 10 years (S6 Fig). Two cases are exemplified (S5 Table), one with 80% recall (sensitivity), and one with maximum precision (positive predicted value). For instance, for the five-year prediction with actual hip fracture incidence of 2.3%, at an 80% sensitivity, the models yielded precisions ranging between 6.7% and 7.9% (S5 Table). Alternatively, maximal precisions for the models ranged from 11.7% to 12.3% at which the corresponding sensitivities ranged from 25.0% to 38.5% (S5 Table).

## Discussion

In this nationwide study of 3,542,647 adults in Sweden aged 50 years and older, an unconditional approach using 139,980 variables was applied to develop and validate, in an untouched holdout cohort, a deep-learning hip fracture prediction model based entirely on national registry data without patient assessment. The best model (FRACTURE-ML), using DeepSurv with 2500 variables, showed excellent performance with a 1-year AUC of 0.89 and sustained accuracy at 5 years (AUC 0.85), representing the most accurate registry-based hip fracture model to date. High performance persisted with a reduced 35-variable model (1-year AUC 0.88), supporting clinical implementation. Cox models performed similarly, showing near-equivalent calibration and discrimination, and all DeepSurv and Cox models demonstrated substantial net benefit across thresholds from 80% sensitivity to 80% specificity.

The fracture prediction tool FREM (fracture risk evaluation model) was developed using Danish hospital diagnoses (*n* = 2,495,339) to predict major osteoporotic fractures and hip fracture. The FREM tool demonstrated high performance in 1-year prediction of hip fractures, particularly in women for which AUC reached 0.87, with slightly worse performance in men with an AUC of 0.85 [13]. Limitations included short-term prediction, no use of machine learning methods for prediction, input variables consisted exclusively on diagnoses, consideration of competing risk of death was not made, and external validation revealed consistent overestimation of hip fracture prediction [14]. Another large study used retrospective electronic health record data from over 1,000,000 individuals and applied a deep learning approach to identify patients with high immediate risk of fracture [27]. While the model achieved an AUC of 0.81 which demonstrated the potential of machine learning methods applied to very large datasets, the utilized black-box approach prevented model interpretability, and without knowledge of clinically relevant factors for fracture risk, and how these are implemented in the model development, credibility for clinical implementation may be questionable. Highly accurate prediction with AUC of 0.89 and 0.91 for women and 0.86 and 0.89 for men was obtained for hip fracture prediction using QFracture and CFracture, respectively, in analyses based on data from general practices in England and Wales, including both diagnosis, treatments and measurements of body mass index and lifestyle factors such as smoking and alcohol use [11,12]. FRAX without BMD yielded somewhat lower AUCs, 0.85 for women and 0.82 for men, using the same cohort data [11]. Although effective, these algorithms are dependent on physical measurements for weight and height, requiring patient interaction, and may not work sufficiently solely on register-based information, and while these variables might have improved FRACTURE-ML even further, they are not available in nationally collected data in Sweden and were therefore not included.

General screening for osteoporosis using the FRAX-tool has been demonstrated to reduce hip fracture numbers in a pooled analysis of randomized trials, but screening is not implemented in clinical practice in most countries [28]. Secondary prevention strategies including coordinator-based FLSs are recommended and implemented in many countries globally [18]. Since FLS is the most commonly used screening tool currently implemented to reduce hip fracture rates and is advocated by Swedish authorities with top priority [22], this approach was used as a comparator to the FRACTURE-ML clinical decision support tool. Although this comparison demonstrated that the proposed hip fracture prediction algorithm identified approximately 7-fold more patients at risk within 2 years and more than 12-fold more at risk within 10 years, with only a marginal reduction in specificity, these different approaches should not be seen as competitive, but rather complementary. Thus, screening for primary prevention using FRACTURE-ML could be used in addition to the already established FLS approach [19] to identify additional risk individuals and prevent a greater proportion of hip fractures than using FLS alone.

A key issue is to select appropriate probability thresholds, which may likely depend on availability of healthcare resources and willingness to invest in follow-up interventions after screening, which will likely vary across the 21 autonomous healthcare regions in Sweden. In our simulation of implementation (Fig 4) in the Swedish population, we either used a high sensitivity or a high specificity scenario, both at 80%, to illustrate the screening consequences. In women, the 80% sensitivity approach would require intervention in 43–64% of the population, depending on age group, whilst the 80% specificity approach would only require intervention in 16%–17%, at the expense of missing more patients with hip fractures. To find an optimal balance which would reduce the number of patients identified for intervention and the numbers of hip fractures missed, a proposed approach would be to implement a system with a variable threshold based on higher specificity in the younger ages and higher sensitivity at older ages.

FRACTURE-ML could be introduced for the primary prevention of hip fracture through a screening-based approach. One potential implementation would be to deploy the model via a national service integrated with registry data needed for FRACTURE-ML calculations. With appropriate consent, patients could access their FRACTURE-ML risk score through a health-service mobile application, an online form, or via a healthcare provider. Individuals identified as having elevated risk could then be offered further evaluation in line with clinical practice, including assessment of physical function and balance, as well as a bone mineral density measurement of the hip and lumbar spine using dual x-ray absorptiometry [29]. These assessments would inform appropriate interventions, such as fall prevention strategies and osteoporosis treatment.

Depending on healthcare setting and reimbursement willingness, different approaches to implement FRACTURE-ML could be used. For example, over a 5-year horizon in a population of 100,000 individuals, applying a FRACTURE-ML screening strategy with 80% sensitivity, which is normally recommended [30], would yield an estimated 2,300 hip fractures, based on the 2.3% 5-year incidence. Of these, 1,840 cases would be correctly identified (80% sensitivity), while 20% (*n* = 460) would remain undetected compared with universal screening. However, only 24,533 individuals would be flagged for further evaluation, corresponding to an approximate 75.5% reduction in the number of individuals requiring further investigation.

The present study has several strengths. It utilized the so far largest cohort ever used to develop a prediction model for hip fracture, using the most comprehensive dataset with a variety of variables routinely available from a large selection of registers, with different levels of detail and historic windows, unconditionally testing a very large number of predictor variables, using machine learning methods, as well as traditional statistical methods. Hip fractures are identified with high accuracy in Swedish registers with PPV of 95% [31]. The resulting AUC of 0.89 is the highest yet achieved for prediction algorithms for hip fracture for models developed on very large datasets without more granular data on BMD, anthropometrics and lifestyle factors which must be obtained by direct patient interaction. This particular tool would allow population-based screening since risk estimates can be provided for the entire population 50 years and older. Furthermore, the algorithm maintained high performance for longer time periods, and after considering the competing risk of death, which currently available automated data-mining-based algorithms have not investigated. Implementation will be facilitated by excellent model performance across different risk levels, by using the simplified 35-variable version requiring less computational effort, and, most importantly, a resource-efficient collection of risk information without the need for unnecessary patient examinations. The statistical reporting is comprehensive with (i) AUC confirming adequate ranking, (ii) precision-recall analyses to assess error rates, (iii) calibration curves to verify that the predicted risks are numerically accurate and (iv) net benefit calculations to show that the model improves real decision-making compared to doing nothing or everything.

Study limitations include lack of patient interview/questionnaire data on lifestyle factors of importance, such as smoking, exercise and alcohol intake. The accuracy and generalizability of FRACTURE-ML must be further validated in other countries. Thus, both external validation in other cohorts and studies testing the implementation of FRACTURE-ML are necessary to successfully evaluate the clinical utility of the tool.

In conclusion, the FRACTURE-ML clinical decision support tool showed excellent hip fracture predictive performance, with an AUC of 0.89 and strong individual-level calibration. Its high accuracy persisted over longer follow-up and after accounting for competing mortality, making it the most accurate hip fracture model tested to date. Implementing FRACTURE-ML could substantially improve efficient identification of high-risk patients, enabling targeted treatment and reducing hip fracture rates.

## Supporting information

S1 Checklist Tripod AI. Completed TRIPOD+AI reporting checklist. The checklist was reproduced from Collins GS, Moons KGM, Dhiman P, and colleagues. The official checklist is available from https://www.tripod-statement.org/wp-content/uploads/2019/12/TRIPODAI_checklist.pdf. The checklist is licensed under the Creative Commons Attribution 4.0 International Licence (CC BY 4.0; https://creativecommons.org/licenses/by/4.0/). The original checklist wording was not modified; manuscript locations and study-specific responses were added by the authors.
(PDF)

S1 Appendix Extended methods. Extended information on study design, data sources, detailed variable descriptions, size of cohorts, discovery phase with overview of variable filtering process, development phase, validation in the holdout cohort, net benefit, clinical implications and statistical analyses
(DOCX)

S2 Appendix Statistical analysis plan. (PDF)

S1 Fig Discovery phase - overview of variable filtering process. Clinically adjacent means variables from same sources with similar codes were analyzed together. The 2% cut-off was selected as it represents the same variable importance as previous fracture last year.
(DOCX)

S2 Fig Loss curves for DeepSurv. The loss functions for training versus validation of DeepSurv 2500 and DeepSurv 35 are presented below. Early stopping was used to monitor the validation loss and prevent potential overfitting of the models.
(PDF)

S3 Fig Calibration plots for hip fracture probabilities in the development cohort. Calibration plots were used to compare the predicted probabilities of hip fracture at 2, 5 and 10 years against the observed events. Original probabilities as well as recalibrated probabilities are presented, with their respective thresholds for high-sensitivity (80% in red) and high-specificity (80% in green). Comparisons were made for Cox 35 and Cox 400 (Panel **A**), DeepSurv 35 and DeepSurv 2500 (Panel **B**) and DeepSurv 35 competing risk (Panel **C**).
(DOCX)

S4 Fig Calibration plots for hip fracture probabilities in the holdout cohort. Calibration plots were used to compare the predicted probabilities of hip fracture at 2, 5 and 10 years against the observed events in the holdout cohort, with their respective thresholds for high-sensitivity (80% in red) and high-specificity (80% in green). Comparisons were made for Cox 35 and Cox 400 (Panel **A**), DeepSurv 2500 and DeepSurv 35 competing (Panel **B**).
(DOCX)

S5 Fig Comparison with the current clinical practice - fracture liaison services. Comparison of hip fracture prediction at 2, 5, and 10 years of the DeepSurv35 and 2500 models to current clinical practice, i.e., Fracture Liaison Service (FLS), represented by any fracture the past year.
(DOCX)

S6 Fig Precision-recall curves for 2, 5 and 10 years prediction. Presentation of precision (=positive predictive value) versus recall (= sensitivity) for hip fracture prediction at 2, 5 and 10 years for Cox 35, Cox 400, DeepSurv 35 and DeepSurv 2500. Calculated in the hold-out cohort (*N* = 354,261). For comparison, hip fracture incidences at 2, 5 and 10 years were 1.0%, 2.3% and 4.0%, respectively.
(DOCX)

S1 Table Cohort characteristics per incident hip fracture (extended data). The table contains a selection of variables from the 139,980 list of candidates (5-year history). SMD = standardized mean difference.
(DOCX)

S2 Table Cohort characteristics per discovery/development/holdout. The table contains a selection of variables from the 139,980 list of candidates split per discovery, development and holdout.
(DOCX)

S3 Table Performance summary of different prediction models after 1, 2, 5 and 10 years. AUC, Area Under Curve; PPV, Positive Predictive Value; NPV, Negative Predictive Value; TN, True Negatives; FP, False Positives; FN, False Negatives; TP, True Positive
(DOCX)

S4 Table Cohort characteristics for the top 35 variables. Cohort characteristics for the 35 most important variables in DeepSurv prediction model. Continuous variables presented with mean ± SD and categoric variables with n (%). Variable importance represents change in AUC (x 1,000,000,000) when randomly permutated, i.e., how much AUC would have changed if the variable had no predictive value. * = missing values treated as separate categories (marital status 3.1%, sickness benefits 3.1% and education level 4.2%)
(DOCX)

S5 Table Values from precision-recall curves for 2, 5 and 10 years prediction. Performance metrics in hold-out cohort (*N* = 354,261) for hip fracture prediction at 2, 5 and 10 years for Cox 35, Cox 400, DeepSurv 35 and DeepSurv 2500 at 80% recall (sensitivity) and maximum precision (positive predictive value). TP, True Positives; FP, False Positives; TN, True Negatives; FN, False Negatives.
(DOCX)

## Abbreviations

AUC: area under the curve
BMD: bone mineral density
FLSs: Fracture Liaison Services
FRACTURE-ML: Fracture Risk Assessment and Classification Using Real-world Evidence and Machine Learning
NAISS: National Academic Infrastructure
NPV: negative predictive values
PPV,: Positive Predictive Value;
ROC: receiver-operating characteristic curves
TRIPOD+AI: Transparent Reporting of a multivariable prediction model for Individual Prognosis Or Diagnosis using machine learning or Artificial Intelligence
UPPMAX: Uppsala Multidisciplinary Centre for Advanced Computational Science
