# Supplementary material for: A clinical decision support tool for accurate hip fracture prediction: A nationwide cohort study
Source: PLoS Med. 2026 Aug 27;23(8):e1005190. doi: 10.1371/journal.pmed.1005190 (PMC13521376; doi:10.1371/journal.pmed.1005190)
Supplement: Supplementary file 2 [file pmed.1005190.s003.pdf]

Statistical Analysis Plan

SUPREME ML FRX

Effective and automated prediction of imminent fractures using available health records and machine learning

March 4, 2025

Henrik Imberg, Oskar Molin, Statistiska Konsultgruppen Sweden AB

Kristian Axelsson, Henrik Litsne, Mattias Lorentzon, Göteborgs Universitet

Updates made by: Konstantinos Konstantinou, Hussnain Khalid, Aldina Pivodic, APNC Sweden AB

Table of Contents

1 Study details 4
1.1 Background 4
1.2 Study objectives 4
1.3 Study design 4
1.4 Study population 4
1.5 Study procedures 4
1.6 Sample size calculations 4
1.6.1 Discovery ..... **Error! Bookmark not defined.**
1.6.2 Development 5
1.6.3 Validation 5
1.7 Compute environment 5
2 Study variables 5
2.1 Outcome variables 5
2.2 Predictors 6
2.2.1 Demographics ..... **Error! Bookmark not defined.**
2.2.2 Medical history 6
3 Statistical methodology 8
3.1 Descriptive statistics 8
3.2 Discovery phase 8
3.3 Model development 8
3.4 Model validation 9
4 Planned outputs 9
4.1 Overall 9
4.2 Discovery phase 9
4.3 Development phase 10
4.4 Validation phase 10
5 References 10

ABBREVIATIONS

ATC	Anatomical therapeutic chemical classification system
AUC	Area under the (receiver operating characteristic) curve
CCI	Charlson comorbidity index
CI	Confidence interval
CIL	Calibration-in-the-large
CS	Calibration slope
DDD	Defined daily code
ICD-10	International classification of diseases and health-related problems
IQR	Interquartile range
LASSO	Least angle shrinkage and selection operator
MOF	Major osteoporotic facture
ROC	Receiver operating characteristic
SD	Standard deviation

1 STUDY DETAILS

1.1 Background

The risk to sustain a hip fracture increases dramatically with age and the proportion of elderly (over 80 years) is estimated by Statistics Sweden to almost double from 5.1% in 2014 to 9.4% in 2060. Hip fractures, are associated with increased morbidity, mortality, and health care costs. Although effective medication exists to strengthen the bone and reduce the risk of fracture, there is extensive undertreatment, and only a fraction of patients eligible for treatment according to Swedish guidelines are actually treated. To prevent fractures, effective methods to identify patients at risk are urgently needed.

1.2 Study objectives

The aim of this study is to use existing national electronic health records and machine learning methods to develop an efficient model to estimate the imminent risk of hip fractures.

1.3 Study design

Register-based retrospective observational study with data collected from multiple national registers, including

i) the National Patient Register with diagnoses and procedures from admissions and outpatient visits to hospitals,

ii) the National Prescribed Drug Register including prescriptions from primary care (thereby indirectly including conditions treated in primary care), and

iii) the longitudinal integrated database for health and labour market studies (LISA) with socioeconomic data.

1.4 Study population

Inclusion criteria were living and registered in Sweden, 50 years or older, alive on 1 January 2011. The patients with osteoporosis medication (<=2 years) were excluded from the study. A total of 3.542.647 subjects were included with a total of 140.131 candidate predictors.

1.5 Study procedures

For each subject, a random baseline (index date) between 1 January 2011 and 31 December 2013 was chosen for inclusion to the study with follow-up until 31 December 2021. The study will be implemented in three stages:

i) **Discovery phase**: using exploratory analyses to identify promising predictors for hip fracture.

ii) **Development phase**: using advanced machine learning techniques to develop multivariable prediction models for fracture, using the predictors identified in the previous step.

iii) **Validation phase**: evaluating the performance of the prediction models developed in the previous steps on new data.

1.6 Sample size calculations

The study population will be subdivided into three sub-cohorts as follows:

i) **Discovery cohort**, comprising n=0.88 millionindividuals (~25% of the total cohort).

ii) **Development cohort**, comprising n= 2.3 million individuals (~65% of the total cohort).

iii) **Validation cohort**, comprising n=0.35 million individuals (~10% of the total cohort).

The sub-cohorts will be constructed using simple random sampling stratified by outcomes and competing events to ensure a similar distribution of outcomes and event-times across the three sub-cohorts.

Sample size calculations for the validation and development phases were performed. Further details are provided below.

1.6.1 Development

Sample size calculations for the model development phase were performed using the three criteria of shrinkage (small amount of overfitting), optimism (small difference between apparent and adjusted R-squared), and precision (precise estimation of the average outcome risk) by Riley *et al* (2020). In particular, the following requirements were set:

i) small overfitting, defined by an expected shrinkage of predictor effects by 10% or less,

ii) small absolute difference of 0.05 in the model's apparent and adjusted Nagelkerke's R-squared value, and

iii) precise estimation (within +/-0.1 percentage points) of the average outcome risk in the population.

Assuming a ten-year fracture incidence of 4% and an AUC of 0.75, up to 7,000 predictors (~5% of all available candidate predictors) can be evaluated with a sample size of at least 1.85 million people.

Calculations were performed by using the R language and environment for statistical computing (R Core Team, Vienna, Austria) version 4.2.3 with the `pmsampsize` package version 1.1.3.

1.6.2 Validation

Sample size calculations for the model validation phase were performed using the three criteria of precision in estimating the observed vs expected event rate (calibration-in-the-large), degree of overfitting (calibration slope), and c-statistic (AUC) by Riley *et al* (2024). In particular, the following requirements were set:

i) precise estimation of the observed vs expected event rate ratio, with an error margin (95% CI halfwidth) of at most ±0.1 units,

ii) precise estimation of the calibration slope, with an error margin of at most ±0.1 units, and

iii) precise estimation of the c-statistic (AUC), with an error margin of at most ±0.1 units.

With a one-year incidence of 0.5% of hip fractures and an AUC of 0.75, a total of 0.3 million people is needed. Thus, approximately 1500 events of hip fractures within one year from baseline will be included in the validation cohort. A higher number of events and consequently higher precision will be obtained for other time frames.

Calculations were performed by using the R language and environment for statistical computing (R Core Team, Vienna, Austria) version 4.2.3 with the `pmvalsampsize` package version 0.1.0.

1.7 Compute environment

Statistical analyses will be performed in a high-performance environment (Bianca) hosted by the Uppsala Multidisciplinary Centre for Advanced Computational Science (UPPMAX) and National Academic Infrastructure for Supercomputing in Sweden (NAIS) for sensitive personal data.

Computations will be performed using distributed data processing and distributed training across multiple compute nodes using Dask-ML. Dask-ML provides scalable machine learning in Python using Dask alongside popular machine learning libraries such as Scikit-Learn and XGBoost.

2 STUDY VARIABLES

2.1 Outcome variables

Time to event of

i) Hip fracture defined as an ICD-10 code of S720-S722 with a code for surgical procedure (NFB, NFC, NFJ), without simultaneous code indicating a revisit (Z09, Z47, Z48). Also, a washout period of five months was used, i.e. a repeat hip fracture diagnosis within five months after the first, was discarded to avoid including codes from revisits. After this, the incident hip fracture variables (1/0 and days to event, censored for death, emigration, end of study (2021-12-31) were created using each patient's random baseline date (between 2011-01-01 and 2013-12-31).

The time to hip fracture outcome, will be evaluated in continuous times using time-to event methodology and specifically presented at 1-, 2-, 5- and 10-years follow-up.

2.2 Predictors

Demographics

- *Age at baseline (years)*
- *Sex (male, female)*
- *Citizenship at birth (Sweden, Nordic country excluding Sweden, EU excluding Nordic countries, Asian, and other including missing)*
- *Education level i.e. highest achieved education at baseline (0–6, ordered categorical from 0 years in school [did not start primary school] to PhD degree), missing treated as separate category (4.2%)*
- *Marital status (unmarried, divorced, widow(er), married, missing (3.1%)) **
- *Urban residency*, i.e. population density ≥200 per km2 in the patient's registered municipality (binary variable, missing treated as non-urban)*
- *Sickness benefits (yes, no, missing (3.1%)) **
  ** Evaluated at 2,5,10 and 13 years prior to baseline.*

Medical history

- Previous fractures (binary) last 1, 2, 5, 10, and 13 years prior to baseline by facture type (three- and four-character ICD-10 codes starting with S02, S12, S22, S32, S42, S52, S62, S72, S82, S92, T02, T08, T10, T12, T142, M485 from both outpatient and inpatient registrations)
- Previous non-skeletal fall injuries(binary) last 1, 2, 5, 10, and 13 years prior to baseline, where non-skeletal fall injury was defined as any fall code (W00-W19) combined with an injury diagnosis (S00-T14) without a fracture during the same occasion.
- Previous illnesses (binary) last 1, 2, 5, 10, and 13 years prior to baseline diagnosed with by ICD-10 code (three- and four-character ICD-10 codes from both outpatient and inpatient registrations)
- Previous medications (binary) last 1, 2 and 5 years prior to baseline, by ATC code (four-, five- and seven-character ATC codes)
- Number of prescriptions of any kind last 1, 2 and 5 years prior to baseline (4 categories: 0, 1-19, 20-73, 74 or more)
- Number of unique ATC-codes prescribed last 1, 2 and 5 years prior to baseline (4 categories: 0, 1-6, 7-13, 14 or more)
- Previous estrogens (binary, ATC-code G03CA) last 1, 2 and 5 years prior to baseline, divided into two groups; systemic or vaginal administration
- Previous osteoporosis medication last 5 years prior to baseline (only one historic window since patients with previous osteoporosis medication last 2 years were not included in study). Specifically, zoledronic acid (M05BA08) and denosumab (M05BX04) were each separated in three different groups depending on if prescribed on (i) osteoporosis indication, (ii) cancer indication (different dose) or (iii) unknown, i.e. registered as outpatient visit with osteoporosis

diagnosis (M80, M81 or M859) and a code for intravenous (DT016) or subcutaneous (DT021) treatment respectively.

- Previous medical procedure (binary) last 1, 2, 5, 10, and 13 years, by procedural code (two and five characters)
- Previous surgical procedure (binary) last 1, 2, 5, 10, and 13 years, by procedural code (three and five characters)
- Hospital usage last 2, 5, 10, and 13 years prior to baseline:

Number of hospitals admissions regardless of length (4 categories: 0, 1, 2 and 3 or more)

Total number of days admitted (4 categories: 0, 1-4, 5-12, 13 or more)

Number of outpatient hospital visits (4 categories: 0, 1-19, 20-73, 74 or more)

- Charlson comorbidity index (CCI) was calculated with four different historic windows (13-10, 13-5, 13-2, 13-0 years prior to baseline) using the weights and ICD-10 codes below resulting in an index which was used as 5 categories (0, 1, 2, 3, 4 or more)

Dementia (1)	F00-F03
Ischemic heart disease (1)	I20-I25
Heart failure (1)	I50
Cerebrovascular disease (1)	I60-I69
Vascular diseases (1)	I70-I79
Chronic pulmonary diseases (1)	J43-J46
Chronic liver disease (1)	K70-K77
Tumor without metastasis (2)	C00-C76, C80, C97
Lymphoma or leukemia (2)	C81-C96
Diabetes (1)	E10-E14
with end organ damage (+1)	E102, E103, E104, E105, E107, E112, E113, E114, E115, E117, H360
Kidney disease (1)	N17-N19
moderate or severe (+1)	N18.2-N18.5
Hemiplegia (2)	G81
Peptic ulcer disease (2)	K25-K27
Metastatic solid tumor (6)	C77-C79

3 STATISTICAL METHODOLOGY

3.1 Descriptive statistics

Descriptive data of participant demographics and outcomes variables will be presented using the mean and standard deviation (SD) for numeric variables. Categorical variables will be presented in numbers and percentages. Descriptive statistics will be presented for the total study cohort and outcomes (fracture, no fracture), and for each sub-cohort (discovery, development, and validation) separately.

The incidence of hip fracture will be estimated using cumulative incidence functions, accounting for censoring and the competing event of death.

3.2 Discovery phase

Candidate predictors for any fracture and hip fracture will be evaluated using the discovery sub-cohort. In the end, at most 7,000 of the top performing predictors (10% of the total predictor set) will be retained for the model development phase.

Predictors for hip fracture will initially be screened using univariable Cox regression analysis adjusted for age and sex, multivariable Cox regression with model selection using LASSO, and XGBoost for time-to-event outcomes. Further details are provided below.

All predictors will be tested one-by-one using age and sex adjusted univariable Cox regression. Univariable models with age interaction will also be investigated. The false discovery rate will be controlled at the 5% level using the Benjamini-Hochberg stepdown procedure, and variables with large standard error estimates will be filtered out. The goal of this step is to select the most prominent features for further analysis.

The selected variables will further be analysed with multivariable Cox regressions with LASSO. In this step, the model selection will be performed in clinically adjacent variable groups (adjusted for age, sex and previous fracture history), and the most prominent variables from each variable group will be selected for further analysis.

Variable selection based on XGboost feature importance will also be investigated. Initially in clinically adjacent groups and then merged with other significant variables. Due to nature of XGBoost, this type of feature selection will consider possible non-linear associations and interactions between the variables.

All candidate predictors will be chosen based on

i) Statistical significance in univariable Cox regression analyses after FDR adjustment

ii) Inclusion in multivariable Cox regression analyses, and

iii) Variable importance using XGBoost.

At most, the 7,000 best performing variables will be used in the subsequent model development phase.

3.3 Model development

A thorough model development process will be implemented on the development data set, utilising the predictors identified in the discovery phase. In the development phase, both traditional and deep learning models will be developed. The following prediction models will primarily be considered:

Traditional models:

- Multivariable Cox regression

- XGBoost for survival outcomes and
- DeepSurv (Deep neural network extension of Cox regression) with and without competing risk adjustment.

Additionally, the following methods may also be considered depending on their applicability for large scale machine learning problems and availability of software implementation for high-performance computing environments:

- Bayesian additive regression trees,
- Optimal scalable decision trees, and
- Optimal risk scoring methods.

Censoring and competing events, and data imbalance issues will be accounted for, as appropriate.

The data will be trained on the full training and the.predictive performance will be evaluated on the test set using Harrell's c-index, Youden cut-off index, sensitivity, specificity, PPV, NPV and ROC curves for hip fracture risk prediction within 1, 2, 5 and 10 years from baseline. Finally, we will investigate the tradeoff between performance and model complexity by also developing reduced models with less covariates. Finally, a final prediction model will be chosen based on the test performance.

The selected models will be refitted on the full development data sets (with identical procedures for hyperparameter tuning) to obtain a final model for validation in the model validation phase while maximising the use of available data.

3.4 Model validation

Model performance will be assessed on the validation sub-cohort to evaluate the performance of the final prediction models derived from the discovery and development phases when tested on new data. Predictive performance will be evaluated in terms of discrimination and calibration as follows:

- Discrimination will be evaluated using Harrell's c-index, Youden cut-off index, sensitivity, specificity, NPV, PPV, and ROC curves for imminent fracture risk prediction within1, 2, 5 and 10 years from baseline for the final model.
- Confusion matrices for the final models.

The calibration of the risk predictions to the observed event rates will be evaluated using the calibration-in-the-large (CIL), calibration slope (CIL), and calibration plots. A value for CIL=0 and CS=1 indicates good calibration. CIL < 0 corresponds to overestimation and CIL > 0 to underestimation of the average outcome risk. CS < 1 corresponds to overfitting (predictions too extreme with high-risk predictions too high and low-risk predictions too low) and CS > 1 to underfitting (predictions that are on average too close to the mean). In addition, data will be split into tertiles according to the predicted risk of fracture and the fracture incidences by risk strata evaluated using cumulative incidence functions, accounting for censoring and competing events.

4 PLANNED OUTPUTS

In the following outputs

4.1 Overall

Table 1: Descriptive statistics of participant demographics and outcomes, overall, by outcome, and by sub-cohort (discovery, development, and validation cohorts).

Figure 1: Cumulative incidence of any fracture and hip fracture during follow-up for the full study cohort.

4.2 Discovery phase

Table 1: Descriptive statistics of participant demographics and outcomes for the discovery cohort.

4.3 Development phase

Table 1: Descriptive statistics of participant demographics and outcomes for the development cohort.

4.4 Validation phase

Table 1: Descriptive statistics of participant demographics and outcomes for the validation cohort.

Table 2: Harrel’s c index, Youden cut-off, specificity, and sensitivity for hip fracture risk prediction within 2, 5 and 10 years from baseline for the final selected model, on the validation cohort.

Figure 1.x: Cumulative incidence of fractures for subjects classified as low-risk (<1st tertile), moderate-risk (≥1st and <2nd tertile) and high-risk (>2nd tertile) according to the final prediction model from the development phase.

Figure 2.x: ROC curves for imminent fracture risk prediction within 1, 2, 5 and 10 years from baseline using the final selected model on the validation cohort.

Figure 3.x: Calibration of predictions for imminent fracture risk prediction within 1, 2,5 and 10 years from baseline using the final selected model on the validation cohort.

Figure 4: Confusion matrices for the final prediction models from the development phase, for the any fracture and hip fracture outcomes, on the validation cohort.

5 REFERENCES

Riley RD, Ensor J, Snell KIE, Harrell FE, Jr., Martin GP, Reitsma JB, et al. Calculating the sample size required for developing a clinical prediction model. *BMJ* 2020;368:m441.

Riley RD, Snell KIE, Archer L, Ensor J, Debray TPA, van Claster B, et al. Evaluation of clinical prediction models (part 3): calculating the sample size required for an external validation study. *BMJ* 2024; 384:e074821.
