# Supplementary material for: A clinical decision support tool for accurate hip fracture prediction: A nationwide cohort study
Source: PLoS Med. 2026 Aug 27;23(8):e1005190. doi: 10.1371/journal.pmed.1005190 (PMC13521376; doi:10.1371/journal.pmed.1005190)
Supplement: Supplementary file 3 [file pmed.1005190.s005.pdf]

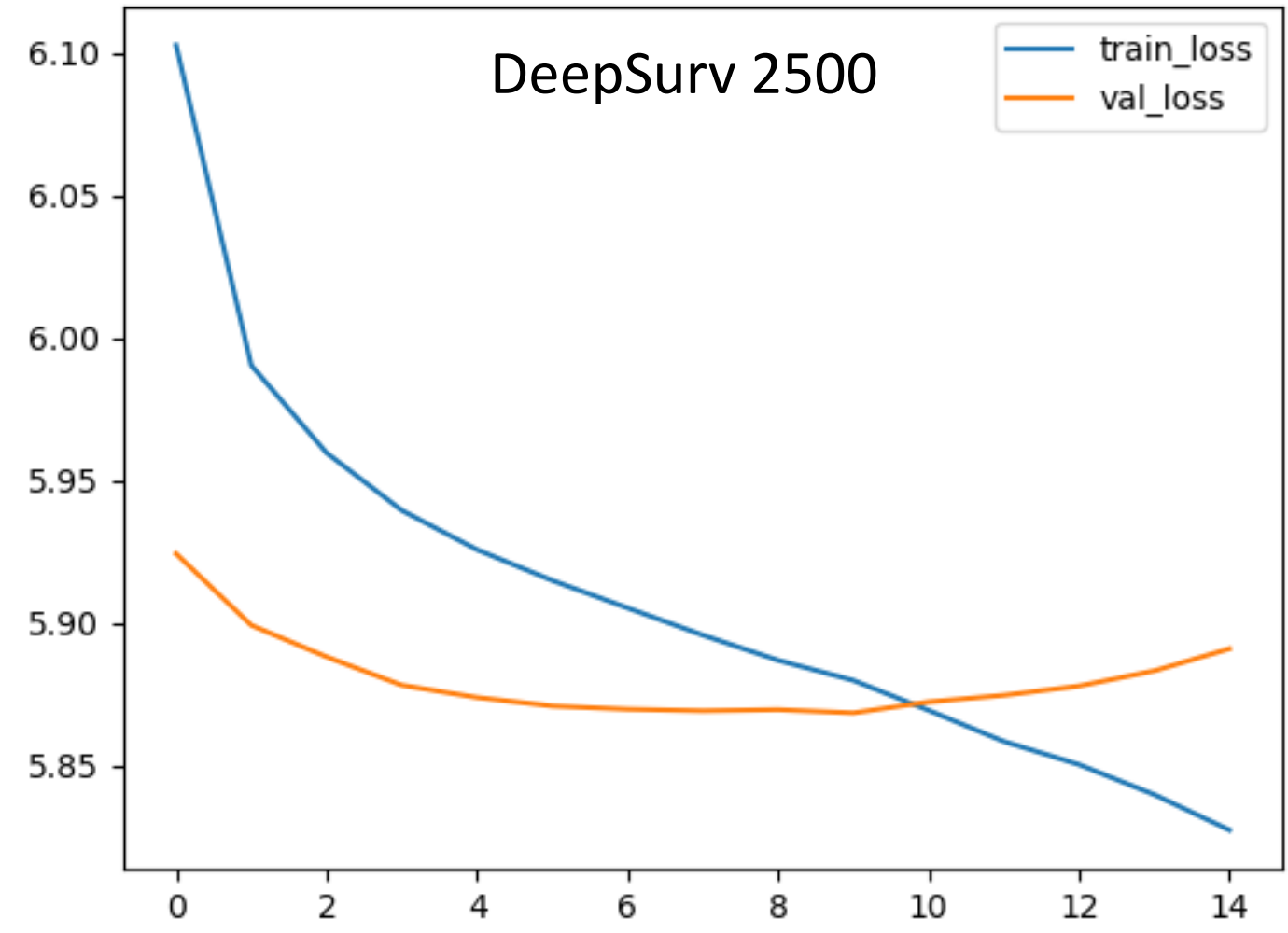
DeepSurv 2500
train_loss
val_loss
6.10
6.05
6.00
5.95
5.90
5.85
0
2
4
6
8
10
12
14

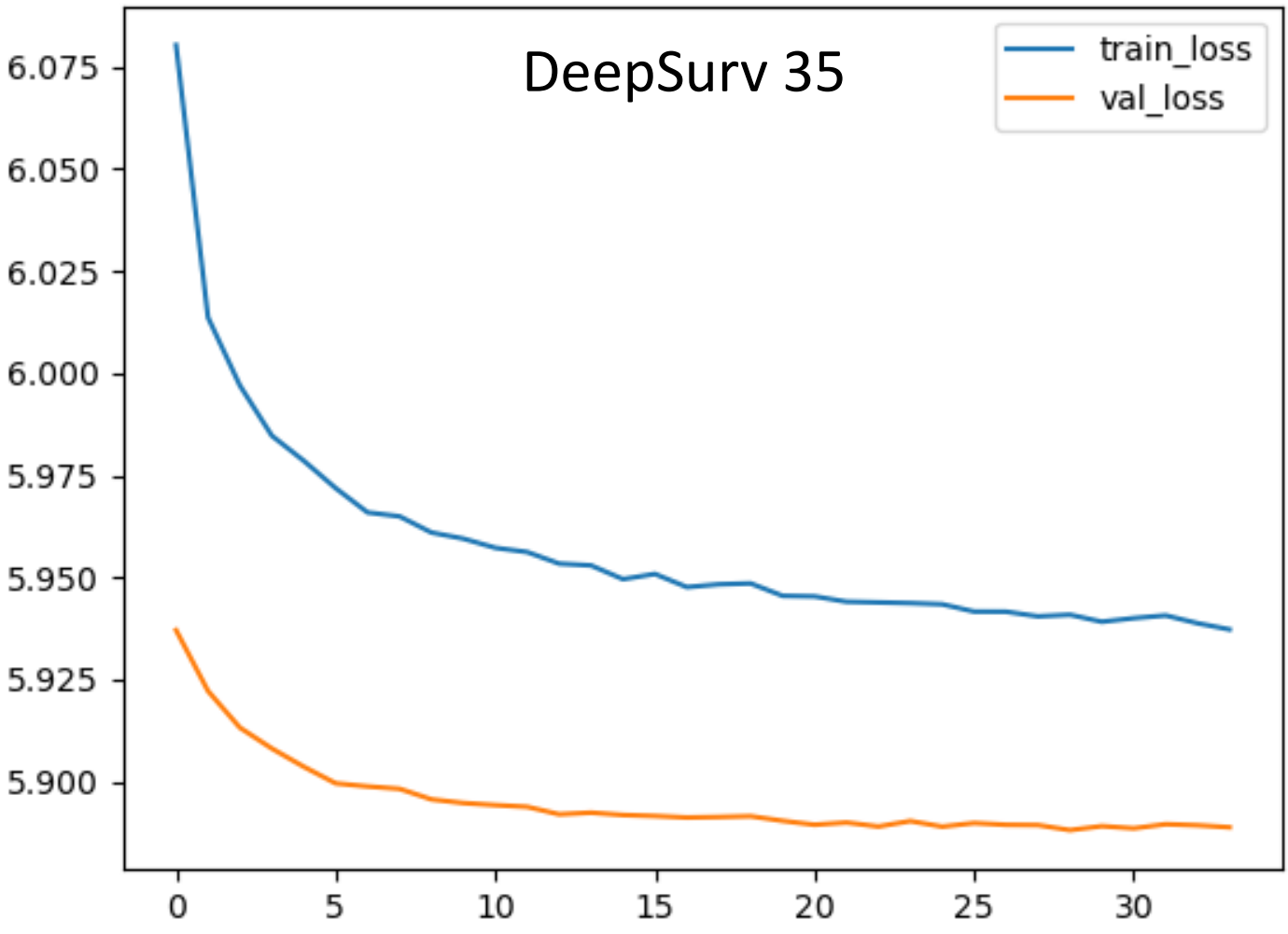
DeepSurv 35
train_loss
val_loss
6.075
6.050
6.025
6.000
5.975
5.950
5.925
5.900
0
5
10
15
20
25
30
