# Supplementary material for: A clinical decision support tool for accurate hip fracture prediction: A nationwide cohort study
Source: PLoS Med. 2026 Aug 27;23(8):e1005190. doi: 10.1371/journal.pmed.1005190 (PMC13521376; doi:10.1371/journal.pmed.1005190)
Supplement: Supplementary file 4 [file pmed.1005190.s002.docx]

**S1 Appendix**

**Extended methods**

**Table of Contents**

[Extended methods	2](#_Toc228307051)

[Study design	2](#_Toc228307052)

[Data sources	2](#_Toc228307053)

[Detailed variable descriptions	2](#_Toc228307054)

[Incident hip fracture	2](#_Toc228307055)

[Prevalent variables	2](#_Toc228307056)

[Demographics	2](#_Toc228307057)

[Medical history	3](#_Toc228307058)

[Size of cohorts	4](#_Toc228307059)

[S1 Fig (copy): Discovery phase - overview of variable filtering process	5](#_Toc228307060)

[Discovery	5](#_Toc228307061)

[Development	6](#_Toc228307062)

[Validation in the holdout cohort	7](#_Toc228307063)

[Net benefit	7](#_Toc228307064)

[Clinical implications	7](#_Toc228307065)

[Statistical analyses	7](#_Toc228307066)

# Extended methods

## Study design

This nationwide cohort study used multiple national registers in Sweden to develop a prediction tool for hip fracture. All Swedish men and women who were born 1981 or earlier and were alive in 2005, were given a random baseline date between 2011-2013. Those aged 50 years or older and alive at baseline were included in the study. Individuals with recent (≤ 2 years) osteoporosis medication (bisfosfonates, denosumab, teriparatide, romosozumab, raloxifene and strontium), were excluded, rendering a population size of 3,542,647 individuals. A broad and unconditional approach was used to create 139,980 baseline variables and possible predictors. Incident hip fracture, i.e. fractured head, neck, trochanter or subtrochanter defined and censored for death (or handled as competing risk), emigration and end of study (Dec 31, 2021). The whole dataset was divided in three separate cohorts for (i) discovery (25%), (ii) development (65%) (of which 15% was used for internal validation for tuning purposes) and (iii) holdout (10%). The risk of hip fracture was evaluated yearly up to ten years using three different methods: traditional statistical models (Cox regression), Deep Learning generalization of the Cox model (DeepSurv), and XGBoost for survival analysis. The study was approved by the Swedish Ethical Review Authority (2022-00796-01).

## Data sources

Information regarding fractures and comorbidities was retrieved from The National Patient Register which includes hospital-based diagnoses from both inpatient and outpatient visits and uses ICD-10 since 1997. Socioeconomic data were retrieved from Statistics Sweden and date of death from Swedish Cause of Death Register. Medication data was collected from The Swedish Prescribed Drug Register, starting July 1st, 2005. In Sweden, all inhabitants are assigned a personal identification number at birth or at the time of immigration, enabling linkage between the registers.

## Detailed variable descriptions

### Incident hip fracture

Incident hip fracture was defined as fractures of the femoral head or neck (S720), trochanter (S721) or subtrochanteric part of the femur (S722) accompanied with a code for surgical procedure (starting with NFB, NFC or NFJ) but not with a simultaneous code for revisit (Z09, Z47, Z48). Also, a washout period of five months was used, i.e. a repeat hip fracture diagnosis within five months after the first, was discarded to avoid including codes from revisits. The follow-up time was censored for death (or handled as competing risk), emigration and end of study (Dec 31, 2021)

### Prevalent variables

A broad unconditional approach with minimal clinical assumptions was used to create baseline variables for demographics, previous diagnoses, fractures, falls, medications, surgical and medical procedures as well as composite variables summarizing morbidity and health care usage. Different levels of detail and different historic windows were defined for all variables. Variables were grouped as follows: 19 demographic variables, 590 fracture-history variables, 5 fall-history variables, 22 composite variables, including the Charlson Comorbidity Index and measures of prescription and hospital use, 61,025 medical-diagnosis variables, 9,794 prescription-history variables, 22,860 medical-procedure variables, and 45,665 surgical-procedure variables. In total, 139,980 variables were investigated. These variables are described in further detail below.

### Demographics

#### Age at baseline (years)

#### Sex (male, female)

#### Citizenship at birth (Sweden, Nordic country excluding Sweden, EU excluding Nordic countries, Asian, and other including missing)

#### Education level i.e. highest achieved education at baseline (0–6, ordered categorical from 0 years in school [did not start primary school] to PhD degree), missing treated as separate category (4.2%)

#### Marital status* (unmarried, divorced, widow(er), married, missing (3.1%))

#### Urban residency*, i.e. population density ≥200 per km^2^ in the patient’s registered municipality (binary variable, missing treated as non-urban)

#### Sickness benefits* (yes, no, missing (3.1%), i.e. any benefits paid by the Swedish Social Insurance Agency when you are unable to work during your normal working hours due to sickness and thus lose income.

#### * Evaluated at baseline and at 2,5,10 and 13 years prior to baseline.

### Medical history

#### Previous fractures (binary) past 1, 2, 5, 10, and 13 years prior to baseline by fracture type (three- and four-character ICD-10 codes starting with S02, S12, S22, S32, S42, S52, S62, S72, S82, S92, T02, T08, T10, T12, T142, M485 from both outpatient and inpatient registrations)

#### Previous non-skeletal fall injuries (binary) the past 1, 2, 5, 10, and 13 years prior to baseline, where non-skeletal fall injury was defined as any fall code (W00-W19) combined with an injury diagnosis (S00-T14) without a fracture during the same occasion.

#### Previous illnesses (binary) the past 1, 2, 5, 10, and 13 years prior to baseline diagnosed with by ICD-10 code (three- and four-character ICD-10 codes from both outpatient and inpatient registrations)

#### Previous medications (binary) the past 1, 2 and 5 years prior to baseline, by ATC code (four-, five- and seven-character ATC codes)

#### Number of prescriptions of any kind the past 1, 2 and 5 years prior to baseline (4 categories: 0, 1-19, 20-73, 74 or more)

#### Number of unique ATC-codes prescribed the past 1, 2 and 5 years prior to baseline (4 categories: 0, 1-6, 7-13, 14 or more)

#### Previous estrogens (binary, ATC-code G03CA) the past 1, 2 and 5 years prior to baseline, divided into two groups; systemic or vaginal administration

#### Previous osteoporosis medication the past 5 years prior to baseline (only one historic window since patients with previous osteoporosis medication the past 2 years were not included in study). Specifically, zoledronic acid (M05BA08) and denosumab (M05BX04) were each separated in three different groups depending on if prescribed on (i) osteoporosis indication, (ii) cancer indication (different dose) or (iii) unknown, i.e. registered as outpatient visit with osteoporosis diagnosis (M80, M81 or M859) and a code for intravenous (DT016) or subcutaneous (DT021) treatment respectively.

#### Previous medical procedure (binary) the past 1, 2, 5, 10, and 13 years, by procedural code (two and five characters)

#### Previous surgical procedure (binary) the past 1, 2, 5, 10, and 13 years, by procedural code (three and five characters)

#### Hospital usage past 2, 5, 10, and 13 years prior to baseline:

##### Number of hospitals admissions regardless of length (4 categories: 0, 1, 2 and 3 or more)

##### Total number of days admitted (4 categories: 0, 1-4, 5-12, 13 or more)

##### Number of outpatient hospital visits (4 categories: 0, 1-19, 20-73, 74 or more)

#### Charlson comorbidity index (CCI) was calculated with four different historic windows (13-10, 13-5, 13-2, 13-0 years prior to baseline) using the weights and ICD-10 codes below resulting in an index which was used as 5 categories (0, 1, 2, 3, 4 or more)

##### Dementia (1) F00-F03

##### Ischemic heart disease (1) I20-I25

##### Heart failure (1) I50

##### Cerebrovascular disease (1) I60-I69

##### Vascular diseases (1) I70-I79

##### Chronic pulmonary diseases (1) J43-J46

##### Chronic liver disease (1) K70-K77

##### Tumour without metastasis (2) C00-C76, C80, C97

##### Lymphoma or leukaemia (2) C81-C96

##### Diabetes (1) E10-E14

##### with end organ damage (+1) E102, E103, E104, E105, E107, E112, E113, E114, E115, E117, H360

##### Kidney disease (1) N17-N19

##### moderate or severe (+1) N18.2-N18.5

##### Hemiplegia (2) G81

##### Peptic ulcer disease (2) K25-K27

##### Metastatic solid tumour (6) C77-C79

## Size of cohorts

Sample size calculations for the *model development* phase were performed using the three criteria of shrinkage (small amount of overfitting), optimism (small difference between apparent and adjusted R-squared), and precision (precise estimation of the average outcome risk).^(1)^ In particular, the following requirements were set:

i) small overfitting, defined by an expected shrinkage of predictor effects by 10% or less,
ii) small absolute difference of 0.05 in the model’s apparent and adjusted Nagelkerke’s R-squared value^(2)^, and
iii) precise estimation (within +/-0.1 percentage points) of the average outcome risk in the population.

Assuming a ten-year fracture incidence of 4% and an AUC of 0.75, up to 7,000 predictors (5% of all available candidate predictors) could be evaluated with a sample size 1.85 million people. As precautionary measure, 2.3 million people (65% of the study population) were included in the development cohort.

Calculations were performed by using the R language and environment for statistical computing (R Core Team, Vienna, Austria) version 4.2.3 with the pmsampsize package version 1.1.3.

Sample size calculations for the *model validation* phase in the holdout cohort were performed using the three criteria of precision in estimating the observed vs expected event rate (calibration-in-the-large), degree of overfitting (calibration slope), and c-statistic (AUC).^(3)^ In particular, the following requirements were set:

i) precise estimation of the observed vs expected event rate ratio, with an error margin (95% CI halfwidth) of at most ±0.1 units,
ii) precise estimation of the calibration slope, with an error margin of at most ±0.1 units, and
iii) precise estimation of the c-statistic (AUC), with an error margin of at most ±0.1 units.

With a one-year incidence of 0.5% of hip fractures and an AUC of 0.75, a total of 0.3 million people was needed. Thus, approximately 1500 events of hip fractures within one year from baseline was included in the holdout cohort. Therefore, 10% of the study population (N = 354,263) were included in the holdout cohort.

Calculations were performed by using the R language and environment for statistical computing (R Core Team, Vienna, Austria) version 4.2.3 with the pmvalsampsize package version 0.1.0.

The remaining 25% of the study population (N = 886,362 individuals) were included in the *discovery cohort*.

## S1 Fig (copy): Discovery phase - overview of variable filtering process

## Discovery

A quarter of the study population was used for discovery (N=886,362). Initially 139,980 candidate variables were considered. To filter out potentially relevant variables, univariable Cox models adjusted for age and sex were performed, with and without interaction with age. The Benjamini-Hochberg^(4)^ stepdown procedure was used to control for multiple testing while maintaining statistical power, resulting in the exclusion of non-significant variables. Variables with large coefficient standard errors (>0.1) were filtered out, leaving 12,960 candidate variables, or 9,431 when not accounting for age-interaction.

The 12,960 variables were further evaluated using XGBoost feature importance scores. Due to memory limitations, this was done in five clinically adjacent groups (socioeconomic, diagnoses, medications, medical procedures and surgical procedures) using XGBoost. All models were adjusted for age, sex and previous fracture history. For each of the five groups, the relative feature importances in relation to the weight for previous fracture occasion 1 year before baseline were calculated. Then, an empirical relative importance threshold of 2% was applied, and all variables with importance scores above that threshold were selected and merged into a new dataset with 4,500 predictors. These variables were fitted in a joint XGBoost model from which 2,500 variables were selected based on their feature importance scores. This selection was based on prior experiments indicating that including more variables yielded minimal performance gains, while substantially increasing computational cost under hardware constraints (e.g., 512GB system memory).

The 9,431 variables from the univariate Cox model screening were further evaluated using multivariable Cox with Lasso penalty. However, as the high multicollinearity led to convergence issues, prevented the fitting of a single Cox model on the full variable dataset. Instead, the variables were analyzed in clinically adjacent variable groups. Given these limitations, subsequent Cox based analyses were conducted on a reduced feature space. To ensure representation across clinical domains, variables were grouped into 40 clinically adjacent groups. From each group, the 10 most important variables (based on XGBoost importance scores) were selected and combined into a joint Cox model (Cox400). This approach ensured that relevant signals from all clinical domains were retained in the final model. A Cox model including all 2,500 variables was not pursued, as even penalized (LASSO) Cox models failed to converge under these conditions. This is consistent with known limitations of regression in settings with strong multicollinearity and large number of predictors. Therefore, Cox analyses were restricted to reduced subsets of variables and used primarily as a baseline modeling approach.

In addition, a more parsimonious model with 35 variables was derived through sequential selection based on XGBoost importance scores. This model was selected because it maintained strong predictive performance while improving feasibility for clinical implementation.

## Development

In the development phase, 65% of the study population (N=2,302,022) was used. All variables selected from the discovery phase were initially used for the training of the predictive models (DeepSurv and XGBoost). As the ten-year incidence of hip fracture is only 4%, subsampling techniques, i.e., subsampling the majority class to match the size of the minority class, were used to balance training and optimize classification performance. The tradeoff between predictive performance and model complexity was investigated by sequentially selecting the most important features using XGBoost feature importance weights. Consequently, the 35 best unique features with respect to timeframe and levels of detail were identified from a bigger pool of predictors (70 variables), and used for training all three predictive models (Cox, DeepSurv and XGBoost). Finally, we developed a DeepSurv model additionally incorporating the competing risk of death into the model. In this model, instead of censoring at the time of death, we assume that the individuals are alive during the whole follow-up period and censored at the end of the follow-up according to the recommendation.^(1)^

The DeepSurv models were tuned by testing key hyperparameter settings, such as hidden layer sizes and dropout rates, to achieve stability and generalization. To avoid overfitting of the model, we tracked the validation loss, i.e. the partial likelihood, for each epoch, and stopped training when the validation loss started to increase (S2 Fig). To handle computational constraints and long queue times, targeted tuning was performed instead of exhaustive grid search. The best configuration showed strong generalization performance. Validation loss monitoring ensured early stopping when overfitting began. The parameters of the Cox model were estimated by maximizing the partial likelihood. Unlike in the discovery phase where we used penalized Cox models (e.g. LASSO regularization) for feature selection, in the development phase no regularization was used. The hyperparameters of the XGBoost model, e.g. max depth, minimum child weight (optimizes the minimum sum of instance weights in the child node) and number of boosting rounds were tuned to optimize performance. To avoid overfitting, we applied early stopping based on the loss of the internal validation set in the development cohort.

In summary, the following models were evaluated:

- DeepSurv (2500 and 35 variables)
- DeepSurv competing (35 variables)
- XGBoost (2500 and 35 variables)
- Cox (400 and 35 variables)

Initial calibration plots in the development cohort revealed a consistent overestimation of hip fracture risk,^(5)^ possibly caused by subsampling, why the predicted risks were recalibrated as follows: “Recalibrated probabilities = Intercept + slope * Original probabilities” where the intercept and slope were estimated using the development cohort data and linear regression, i.e. minimizing the sum of the squared errors. In some subgroups (e.g. young healthy people) the recalibrated probabilities became negative due to a negative intercept. These probabilities were adjusted to zero. Then, the recalibrated probabilities from the development cohort were used to estimate overall and subgroup specific 80% sensitivity and 80% specificity cutoffs. This was performed for the Cox models and DeepSurv models (S5 Fig). For the XGBoost models, it was not possible to calculate individual risks.

## Validation in the holdout cohort

Several models were tested, with prediction compared to observed, for different time periods and presented with ROC curves, AUC, sensitivity, specificity, PPV, NPV, calibration plots together with 95% bootstrapped confidence intervals, and confusion matrices. For classification, the predicted fracture probabilities of the individuals in the discovery cohort were used to select Youden cutoffs for every year. These cutoffs were then used on the predicted fractured probabilities of the holdout cohort to make predictions for evaluating model performance.

For evaluating the DeepSurv and Cox models, we had to filter out 2 individuals from the holdout cohort due to missing data. As a result, the models were evaluated on 354,261 individuals. On the other hand, as the XGBoost model can predict even in the presence of missing data, it was evaluated on the full holdout cohort (N=354,263). The importance of the 35 variables of the reduced DeepSurv method was investigated using permutation importance with change in AUC as a statistic.

Calibration plots were performed to compare the predicted probabilities of hip fracture at 2, 5 and 10 years with the observed events for DeepSurv 35 (Figure 3), Cox 35 and Cox 400 (S4 Fig Panel A) and DeepSurv 35 and 2500 (S4 Fig Panel B).

## Net benefit

To quantify the clinical usefulness of the developed models (DeepSurv 2500, DeepSurv 35, Cox 400, Cox 35), net benefit was calculated as (TP – FP x PB/(1-PB))/N where TP=true positives, FP=false positives, PB=threshold probability, N= observations. Net benefit was then compared to screen all, screen none and screening using Fracture Liaison Services. Default strategies screen all and screen none are represented as dark grey dotted lines. Threshold probabilities representing 80% sensitivity (red) and 80% specificity (blue) are marked for Deep Surv 35.

## Clinical implications

To demonstrate the clinical implications, the hip fracture risk within 2 years for selected health profiles and age groups, as predicted by the model for women and men were presented. Also, two different cases, high-sensitivity and high-specificity, were applied and presented to exemplify how different implementation approaches affect thresholds and potential health care budget. Finally, a comparison of best model was made with the current model in clinical practice, i.e. Fracture Liaison Service (FLS) represented by any fracture the past year.

## Statistical analyses

Descriptive baseline statistics for most common diagnoses, medications, medical and surgical procedures, as well as socioeconomical and previous fracture history variables are presented in terms of counts with percentage for categorical variables and averages with standard deviations (SD) for continuous variables. Statistical analyses were performed using Python and popular machine learning libraries such as Pycox, Scikit-Learn, and XGBoost. Computations were performed using distributed data processing and distributed training across multiple compute nodes in a high-performance environment (Bianca) hosted by Uppsala Multidisciplinary Centre for Advanced Computational Science (UPPMAX) and National Academic Infrastructure (NAIS) for sensitive personal data.

**References**

1.	Riley RD, Ensor J, Snell KIE, Harrell FE, Jr., Martin GP, Reitsma JB, et al. Calculating the sample size required for developing a clinical prediction model. Bmj. Mar 18 2020;368:m441. Epub 20200318.

2.	Nagelkerke NJD. A note on a general definition of the coefficient of determination. Biometrika. 1991;78(3):691-2.

3.	Riley RD, Snell KIE, Archer L, Ensor J, Debray TPA, van Calster B, et al. Evaluation of clinical prediction models (part 3): calculating the sample size required for an external validation study. Bmj. Jan 22 2024;384:e074821. Epub 20240122.

4.	Benjamini Y, Hochberg Y. Controlling the False Discovery Rate: A Practical and Powerful Approach to Multiple Testing. Journal of the Royal Statistical Society: Series B (Methodological). 2018;57(1):289-300.

5.	Guo, C., Pleiss, G., Sun, Y., & Weinberger, K. Q. (2017, July). On calibration of modern neural networks. In International conference on machine learning (pp. 1321-1330). PMLR.
