# Supplementary material for: A clinical decision support tool for accurate hip fracture prediction: A nationwide cohort study
Source: PLoS Med. 2026 Aug 27;23(8):e1005190. doi: 10.1371/journal.pmed.1005190 (PMC13521376; doi:10.1371/journal.pmed.1005190)
Supplement: Supplementary file 5 [file pmed.1005190.s004.docx]

## S1 Fig: Discovery phase - overview of variable filtering process

Overview of variable selection process in discovery phase. Clinically adjacent means variables from same sources with similar codes were analyzed together. The 2% cut-off was selected as it represents the same variable importance as previous fracture last year.
