# Supplementary material for: A clinical decision support tool for accurate hip fracture prediction: A nationwide cohort study
Source: PLoS Med. 2026 Aug 27;23(8):e1005190. doi: 10.1371/journal.pmed.1005190 (PMC13521376; doi:10.1371/journal.pmed.1005190)
Supplement: Supplementary file 6 [file pmed.1005190.s006.docx]

# S3 Fig. Calibration plots for hip fracture probabilities in the development cohort

Calibration plots were used to compare the predicted probabilities of hip fracture at 2, 5 and 10 years against the observed events. Original probabilities as well as recalibrated probabilities are presented, with their respective thresholds for high-sensitivity (80% in red) and high-specificity (80% in green).

# Panel A Cox 35 and Cox 400

# Panel B: DeepSurv 35 and DeepSurv 2500

# Panel C: DeepSurv 35 competing risk, accounting for competing risk of death
