# Supplementary material for: A clinical decision support tool for accurate hip fracture prediction: A nationwide cohort study
Source: PLoS Med. 2026 Aug 27;23(8):e1005190. doi: 10.1371/journal.pmed.1005190 (PMC13521376; doi:10.1371/journal.pmed.1005190)
Supplement: Supplementary file 7 [file pmed.1005190.s007.docx]

# S4 Fig. Calibration plots for hip fracture probabilities in the holdout cohort

Calibration plots were used to compare the predicted probabilities of hip fracture at 2, 5 and 10 years against the observed events in the holdout cohort, with their respective thresholds for high-sensitivity (80% in red) and high-specificity (80% in green).

# Panel A Cox 35 and Cox 400

# Panel B DeepSurv 2500 and DeepSurv 35 competing
