# Supplementary material for: A clinical decision support tool for accurate hip fracture prediction: A nationwide cohort study
Source: PLoS Med. 2026 Aug 27;23(8):e1005190. doi: 10.1371/journal.pmed.1005190 (PMC13521376; doi:10.1371/journal.pmed.1005190)
Supplement: Supplementary file 8 [file pmed.1005190.s008.docx]

# S5 Fig. Comparison with the current clinical practice - fracture liaison services

Comparison of hip fracture prediction at 2, 5, 10 years of the DeepSurv35 and 2500 models to current clinical practice, i.e. Fracture Liaison Service (FLS), represented by any fracture the past year.
