# Supplementary material for: A clinical decision support tool for accurate hip fracture prediction: A nationwide cohort study
Source: PLoS Med. 2026 Aug 27;23(8):e1005190. doi: 10.1371/journal.pmed.1005190 (PMC13521376; doi:10.1371/journal.pmed.1005190)
Supplement: Supplementary file 9 [file pmed.1005190.s009.docx]

# S6 Fig. Precision-recall curves for 2, 5 and 10 years prediction

Presentation of precision (=positive predictive value) vs recall (= sensitivity) for hip fracture prediction at 2, 5 and 10 years for Cox 35, Cox 400, DeepSurv 35 and DeepSurv 2500. Calculated in the hold-out cohort (N= 354,261). For comparison, hip fracture incidences at 2, 5 and 10 years were 1.0%, 2.3% and 4.0% respectively.
