# Supplementary material for: A clinical decision support tool for accurate hip fracture prediction: A nationwide cohort study
Source: PLoS Med. 2026 Aug 27;23(8):e1005190. doi: 10.1371/journal.pmed.1005190 (PMC13521376; doi:10.1371/journal.pmed.1005190)
Supplement: Supplementary file 10 [file pmed.1005190.s010.docx]

## S1 Table. Cohort characteristics per incident hip fracture (extended data)

|  | **ALL** | **Incident hip fracture** | | **SMD** |
| --- | --- | --- | --- | --- |
|  |  | **yes** | **no** |  |
|  | **N=3,542,647** | **N=142,327** | **N=3,400,320** |  |
| Top 10 most common diagnoses — no. (%) | |  |  |  |
| Hypertension (I10) | 585,736 (16.5) | 40,707 (28.6) | 545,029 (16.0) | 0.34 |
| Follow-up visit (Z09) | 505,574 (14.3) | 30,990 (21.8) | 474,584 (14.0) | 0.22 |
| Senile cataract (H25) | 343,846 (9.7) | 32,436 (22.8) | 311,410 (9.2) | 0.46 |
| Routine examination (Z01) | 259,733 (7.3) | 12,053 (8.5) | 247,680 (7.3) | 0.05 |
| Medical observation (Z03) | 233,995 (6.6) | 13,351 (9.4) | 220,644 (6.5) | 0.12 |
| Presence of functional implant (Z96) | 233,920 (6.6) | 21,047 (14.8) | 212,873 (6.3) | 0.34 |
| Pain in throat and chest (R07) | 225,198 (6.4) | 10,930 (7.7) | 214,268 (6.3) | 0.06 |
| Abdominal and pelvic pain (R10) | 221,104 (6.2) | 10,163 (7.1) | 210,941 (6.2) | 0.04 |
| Other soft tissue disorders (M79) | 207,656 (5.9) | 10,080 (7.1) | 197,576 (5.8) | 0.05 |
| Type 2 diabetes mellitus (E11) | 205,161 (5.8) | 13,756 (9.7) | 191,405 (5.6) | 0.17 |
| Top 10 most common medications — no. (%) | |  |  |  |
| Paracetamol (N02BE01) | 1,214,022 (34.3) | 70,140 (49.3) | 1,143,882 (33.6) | 0.33 |
| Diclofenac (M01AB05) | 1,035,106 (29.2) | 34,726 (24.4) | 1,000,380 (29.4) | -0.11 |
| Phenoxymethylpenicillin (J01CE02) | 1,033,134 (29.2) | 38,666 (27.2) | 994,468 (29.2) | -0.05 |
| Simvastatin (C10AA01) | 810,909 (22.9) | 43,419 (30.5) | 767,490 (22.6) | 0.19 |
| Omeprazole (A02BC01) | 809,266 (22.8) | 41,569 (29.2) | 767,697 (22.6) | 0.16 |
| Acetylsalicylic acid (B01AC06) | 748,277 (21.1) | 53,652 (37.7) | 694,625 (20.4) | 0.42 |
| Doxycycline (J01AA02) | 624,003 (17.6) | 25,106 (17.6) | 598,897 (17.6) | 0.00 |
| Enalapril (C09AA02) | 595,691 (16.8) | 33,303 (23.4) | 562,388 (16.5) | 0.18 |
| Metoprolol (C07AB02) | 568,396 (16.0) | 35,544 (25.0) | 532,852 (15.7) | 0.25 |
| Tramadol (N02AX02) | 513,768 (14.5) | 26,822 (18.8) | 486,946 (14.3) | 0.13 |
| Top 10 most common medical procedures — no. (%) | |  |  |  |
| Transthoracic echo, short (AF020) | 137,332 (3.9) | 8,227 (5.8) | 129,105 (3.8) | 0.10 |
| Vaginal ultrasound (AL003) | 133,286 (3.8) | 4,957 (3.5) | 128,329 (3.8) | -0.02 |
| Exercise electrocardiogram (AF012) | 129,248 (3.6) | 4,998 (3.5) | 124,250 (3.7) | -0.01 |
| Coronary x-ray (AF037) | 119,051 (3.4) | 6,028 (4.2) | 113,023 (3.3) | 0.05 |
| Preventive discussion (DV030) | 117,624 (3.3) | 5,874 (4.1) | 111,750 (3.3) | 0.05 |
| Meeting with doctor (XS900) | 117,340 (3.3) | 5,851 (4.1) | 111,489 (3.3) | 0.05 |
| Transthoracic echo, full (AF021) | 110,801 (3.1) | 6,725 (4.7) | 104,076 (3.1) | 0.10 |
| Telemetry monitoring (AF063) | 108,513 (3.1) | 7,635 (5.4) | 100,878 (3.0) | 0.14 |
| Electrocardiogram (AF022) | 107,984 (3.0) | 7,099 (5.0) | 100,885 (3.0) | 0.12 |
| Telephone counseling (XS012) | 94,035 (2.7) | 4,537 (3.2) | 89,498 (2.6) | 0.03 |
| Top 10 most common surgical procedures — no. (%) | |  |  |  |
| Cataract surgery (CJE20) | 206,020 (5.8) | 19,005 (13.4) | 187,015 (5.5) | 0.34 |
| Gastroscopy (UJD02) | 151,671 (4.3) | 9,278 (6.5) | 142,393 (4.2) | 0.12 |
| Cystoscopy (UKC02) | 121,425 (3.4) | 7,276 (5.1) | 114,149 (3.4) | 0.10 |
| Right side (ZXA00) | 117,659 (3.3) | 7,696 (5.4) | 109,963 (3.2) | 0.12 |
| Left side (ZXA05) | 112,284 (3.2) | 7,474 (5.3) | 104,810 (3.1) | 0.12 |
| Transanal ultrasonography (XJH00) | 102,135 (2.9) | 4,603 (3.2) | 97,532 (2.9) | 0.02 |
| Biopsy of skin (TQX00) | 88,715 (2.5) | 5,408 (3.8) | 83,307 (2.4) | 0.09 |
| Gastroscopy with biopsy (UJD05) | 86,181 (2.4) | 5,025 (3.5) | 81,156 (2.4) | 0.07 |
| Colonoscopy (UJF32) | 84,937 (2.4) | 4,902 (3.4) | 80,035 (2.4) | 0.07 |
| Cryotherapy in head-neck (QAA50) | 79,173 (2.2) | 5,603 (3.9) | 73,570 (2.2) | 0.12 |

The table contains a selection of variables from the 139,980 list of candidates (5-year history)

SMD=standardized mean difference
