# Supplementary material for: A clinical decision support tool for accurate hip fracture prediction: A nationwide cohort study
Source: PLoS Med. 2026 Aug 27;23(8):e1005190. doi: 10.1371/journal.pmed.1005190 (PMC13521376; doi:10.1371/journal.pmed.1005190)
Supplement: Supplementary file 11 [file pmed.1005190.s011.docx]

## S2 Table. Cohort characteristics per discovery/development/holdout – part 1

|  | **All** | **Discovery** | **Development** | **Holdout** |
| --- | --- | --- | --- | --- |
|  | **N=3,542,647** | **N=886,362** | **N=2,302,022** | **N=354,263** |
| Age, yrs — mean (SD) | 65.9 (11.0) | 65.7 (11.0) | 66.0 (11.0) | 66.0 (11.0) |
| Female Sex — no. (%) | 1,805,997 (51.0) | 450,189 (50.8) | 1,174,877 (51.0) | 180,931 (51.1) |
| Urban residency — no. (%) | 815,971 (23.0) | 199,857 (22.5) | 534,320 (23.2) | 81,794 (23.1) |
| Sickness benefits — no. (%) | 208,633 (5.9) | 50,624 (5.7) | 136,914 (5.9) | 21,095 (6.0) |
| Education, < 9 yrs of schooling — no. (%) | 660,938 (18.7) | 160,778 (18.1) | 433,173 (18.8) | 66,987 (18.9) |
| Marital status — no. (%) |  |  |  |  |
| unmarried | 535,448 (15.1) | 130,827 (14.8) | 350,804 (15.2) | 53,817 (15.2) |
| divorced | 621,262 (17.5) | 151,410 (17.1) | 407,283 (17.7) | 62,569 (17.7) |
| widow(er) | 417,372 (11.8) | 102,017 (11.5) | 273,195 (11.9) | 42,160 (11.9) |
| married | 1,858,377 (52.5) | 452,769 (51.1) | 1,218,060 (52.9) | 187,548 (52.9) |
| Citizenship at birth — no. (%) |  |  |  |  |
| Swedish | 2,985,555 (84.3) | 727,713 (82.1) | 1,956,927 (85.0) | 300,915 (84.9) |
| Nordic | 183,304 (5.2) | 48,488 (5.5) | 116,675 (5.1) | 18,141 (5.1) |
| European | 204,704 (5.8) | 56,886 (6.4) | 128,156 (5.6) | 19,662 (5.6) |
| Asian | 107,046 (3.0) | 34,313 (3.9) | 62,910 (2.7) | 9,823 (2.8) |
| Other | 62,038 (1.8) | 18,962 (2.1) | 37,354 (1.6) | 5,722 (1.6) |
| Previous event — no. (%) |  |  |  |  |
| Fracture, past yr | 85,845 (2.4) | 21,052 (2.4) | 56,220 (2.4) | 8,573 (2.4) |
| Fracture, past 10 yrs | 476,366 (13.4) | 115,753 (13.1) | 312,911 (13.6) | 47,702 (13.5) |
| Fall injury, past yr | 56,833 (1.6) | 13,854 (1.6) | 37,124 (1.6) | 5,855 (1.7) |
| Fall injury, past 10 yrs | 333,111 (9.4) | 81,125 (9.2) | 217,998 (9.5) | 33,988 (9.6) |
| Osteoporosis medication past 2- 5 yrs — no. (%) | 55,229 (1.6%) | 13,302 (1.5%) | 36,268 (1.6%) | 5,659 (1.6%) |
| Systemic estrogens, past 5 years — no. (%) | 128,780 (3.6%) | 31,383 (3.5%) | 84,298 (3.7%) | 13,099 (3.7%) |
| Charlson Comorbidity Index — no. (%) |  |  |  |  |
| 0 | 2,377,896 (67.1) | 603,226 (68.1) | 1,537,969 (66.8) | 236,701 (66.8) |
| 1 | 416,840 (11.8) | 101,419 (11.4) | 273,139 (11.9) | 42,282 (11.9) |
| 2 | 426,323 (12.0) | 103,911 (11.7) | 279,363 (12.1) | 43,049 (12.2) |
| 3 | 153,047 (4.3) | 37,142 (4.2) | 100,515 (4.4) | 15,390 (4.3) |
| ≥4 | 168,541 (4.8) | 40,664 (4.6) | 111,036 (4.8) | 16,841 (4.8) |
| Admissions past 5 yrs — no. (%) |  |  |  |  |
| 0 | 2,280,174 (64.4) | 579,976 (65.4) | 1,473,531 (64.0) | 226,667 (64.0) |
| 1 | 589,901 (16.7) | 143,233 (16.2) | 386,972 (16.8) | 59,696 (16.9) |
| 2 | 266,806 (7.5) | 64,693 (7.3) | 175,174 (7.6) | 26,939 (7.6) |
| ≥3 | 405,766 (11.5) | 98,460 (11.1) | 266,345 (11.6) | 40,961 (11.6) |
| Different prescriptions past 5 yrs — no. (%) |  |  |  |  |
| 0 | 248,741 (7.0) | 82,574 (9.3) | 143,940 (6.3) | 22,227 (6.3) |
| 1-6 | 1,098,688 (31.0) | 270,779 (30.5) | 717,753 (31.2) | 110,156 (31.1) |
| 7-13 | 1,038,056 (29.3) | 252,304 (28.5) | 681,253 (29.6) | 104,499 (29.5) |
| 14 or more | 1,157,162 (32.7) | 280,705 (31.7) | 759,076 (33.0) | 117,381 (33.1) |

The table contains a selection of variables from the 139,980 list of candidates split per discovery, development and holdout

## S2 Table. Cohort characteristics per discovery/development/holdout – part 2

|  | **All** | **Discovery** | **Development** | **Holdout** |
| --- | --- | --- | --- | --- |
|  | **N=3,542,647** | **N=886,362** | **N=2,302,022** | **N=354,263** |
| Top 10 most common diagnoses — no. (%) |  |  |  |  |
| Hypertension (I10) | 585,736 (16.5) | 142,583 (16.1) | 384,078 (16.7) | 59,075 (16.7) |
| Follow-up visit (Z09) | 505,574 (14.3) | 122,285 (13.8) | 332,119 (14.4) | 51,170 (14.4) |
| Senile cataract (H25) | 343,846 (9.7) | 83,407 (9.4) | 225,881 (9.8) | 34,558 (9.8) |
| Routine examination (Z01) | 259,733 (7.3) | 63,374 (7.1) | 170,174 (7.4) | 26,185 (7.4) |
| Medical observation (Z03) | 233,995 (6.6) | 57,012 (6.4) | 153,043 (6.6) | 23,940 (6.8) |
| Presence of functional implant (Z96) | 233,920 (6.6) | 56,680 (6.4) | 153,470 (6.7) | 23,770 (6.7) |
| Pain in throat and chest (R07) | 225,198 (6.4) | 54,538 (6.2) | 147,623 (6.4) | 23,037 (6.5) |
| Abdominal and pelvic pain (R10) | 221,104 (6.2) | 53,806 (6.1) | 144,591 (6.3) | 22,707 (6.4) |
| Other soft tissue disorders (M79) | 207,656 (5.9) | 50,397 (5.7) | 136,293 (5.9) | 20,966 (5.9) |
| Type 2 diabetes mellitus (E11) | 205,161 (5.8) | 49,886 (5.6) | 134,727 (5.9) | 20,548 (5.8) |
| Top 10 most common medications — no. (%) |  |  |  |  |
| Paracetamol (N02BE01) | 1,214,022 (34.3) | 294,921 (33.3) | 796,505 (34.6) | 122,596 (34.6) |
| Diclofenac (M01AB05) | 1,035,106 (29.2) | 252,646 (28.5) | 677,762 (29.4) | 104,698 (29.6) |
| Phenoxymethylpenicillin (J01CE02) | 1,033,134 (29.2) | 251,337 (28.4) | 677,218 (29.4) | 104,579 (29.5) |
| Simvastatin (C10AA01) | 810,909 (22.9) | 197,249 (22.3) | 532,335 (23.1) | 81,325 (23.0) |
| Omeprazole (A02BC01) | 809,266 (22.8) | 196,474 (22.2) | 530,733 (23.1) | 82,059 (23.2) |
| Acetylsalicylic acid (B01AC06) | 748,277 (21.1) | 181,772 (20.5) | 490,971 (21.3) | 75,534 (21.3) |
| Doxycycline (J01AA02) | 624,003 (17.6) | 151,561 (17.1) | 409,325 (17.8) | 63,117 (17.8) |
| Enalapril (C09AA02) | 595,691 (16.8) | 145,022 (16.4) | 390,869 (17.0) | 59,800 (16.9) |
| Metoprolol (C07AB02) | 568,396 (16.0) | 138,112 (15.6) | 372,821 (16.2) | 57,463 (16.2) |
| Tramadol (N02AX02) | 513,768 (14.5) | 124,763 (14.1) | 336,829 (14.6) | 52,176 (14.7) |
| Top 10 most common medical procedures — no. (%) | |  |  |  |
| Transthoracic echocardiography, short (AF020) | 137,332 (3.9) | 33,416 (3.8) | 90,007 (3.9) | 13,909 (3.9) |
| Vaginal ultrasound (AL003) | 133,286 (3.8) | 32,613 (3.7) | 87,204 (3.8) | 13,469 (3.8) |
| Exercise electrocardiogram (AF012) | 129,248 (3.6) | 31,449 (3.5) | 84,584 (3.7) | 13,215 (3.7) |
| Coronary x-ray (AF037) | 119,051 (3.4) | 28,845 (3.3) | 78,097 (3.4) | 12,109 (3.4) |
| Preventive discussion (DV030) | 117,624 (3.3) | 28,653 (3.2) | 77,340 (3.4) | 11,631 (3.3) |
| Meeting with doctor (XS900) | 117,340 (3.3) | 28,386 (3.2) | 77,333 (3.4) | 11,621 (3.3) |
| Transthoracic echocardiography, full (AF021) | 110,801 (3.1) | 26,758 (3.0) | 72,792 (3.2) | 11,251 (3.2) |
| Telemetry monitoring (AF063) | 108,513 (3.1) | 26,392 (3.0) | 70,995 (3.1) | 11,126 (3.1) |
| Electrocardiogram (AF022) | 107,984 (3.0) | 26,115 (2.9) | 70,884 (3.1) | 10,985 (3.1) |
| Telephone counseling (XS012) | 94,035 (2.7) | 22,826 (2.6) | 61,810 (2.7) | 9,399 (2.7) |
| Top 10 most common surgical procedures — no. (%) | |  |  |  |
| Cataract surgery (CJE20) | 206,020 (5.8) | 49,920 (5.6) | 135,386 (5.9) | 20,714 (5.8) |
| Gastroscopy (UJD02) | 151,671 (4.3) | 36,750 (4.1) | 99,529 (4.3) | 15,392 (4.3) |
| Cystoscopy (UKC02) | 121,425 (3.4) | 29,573 (3.3) | 79,659 (3.5) | 12,193 (3.4) |
| Right side (ZXA00) | 117,659 (3.3) | 28,429 (3.2) | 77,264 (3.4) | 11,966 (3.4) |
| Left side (ZXA05) | 112,284 (3.2) | 27,181 (3.1) | 73,540 (3.2) | 11,563 (3.3) |
| Transanal ultrasonography (XJH00) | 102,135 (2.9) | 24,867 (2.8) | 66,995 (2.9) | 10,273 (2.9) |
| Biopsy of skin (TQX00) | 88,715 (2.5) | 21,563 (2.4) | 58,049 (2.5) | 9,103 (2.6) |
| Gastroscopy with biopsy (UJD05) | 86,181 (2.4) | 20,978 (2.4) | 56,527 (2.5) | 8,676 (2.4) |
| Colonoscopy (UJF32) | 84,937 (2.4) | 20,803 (2.3) | 55,557 (2.4) | 8,577 (2.4) |
| Cryotherapy in head-neck (QAA50) | 79,173 (2.2) | 19,290 (2.2) | 51,885 (2.3) | 7,998 (2.3) |

The table contains a selection of variables from the 139,980 list of candidates split per discovery, development and holdout (five-year history)
