# Supplementary material for: A clinical decision support tool for accurate hip fracture prediction: A nationwide cohort study
Source: PLoS Med. 2026 Aug 27;23(8):e1005190. doi: 10.1371/journal.pmed.1005190 (PMC13521376; doi:10.1371/journal.pmed.1005190)
Supplement: Supplementary file 12 [file pmed.1005190.s012.docx]

# S3 Table. Performance summary of different prediction models after 1, 2, 5 and 10 years

| **Analysis** | **Yr** | **AUC** | **Sensitivity** | **Specificity** | **PPV** | **NPV** | **TN** | **FP** | **FN** | **TP** |
| --- | --- | --- | --- | --- | --- | --- | --- | --- | --- | --- |
| DeepSurv 2500 | 1 | 0.885 (0.879-0.891) | 0.828 (0.813-0.846) | 0.809 (0.808-0.811) | 0.022 (0.021-0.023) | 0.999 (0.999-0.999) | 285,215 | 67,218 | 315 | 1,513 |
| DeepSurv 2500 | 2 | 0.877 (0.871-0.881) | 0.836 (0.822-0.851) | 0.792 (0.791-0.793) | 0.038 (0.037-0.039) | 0.998 (0.998-0.998) | 277,862 | 72,947 | 568 | 2,884 |
| DeepSurv 2500 | 5 | 0.853 (0.849-0.856) | 0.832 (0.825-0.839) | 0.752 (0.750-0.753) | 0.072 (0.071-0.074) | 0.995 (0.995-0.995) | 260,228 | 86,007 | 1,345 | 6,681 |
| DeepSurv 2500 | 10 | 0.815 (0.813-0.818) | 0.808 (0.803-0.814) | 0.707 (0.706-0.709) | 0.103 (0.102-0.105) | 0.989 (0.988-0.989) | 240,526 | 99,552 | 2,722 | 11,461 |
| DeepSurv 35 | 1 | 0.878 (0.871-0.885) | 0.823 (0.808-0.841) | 0.794 (0.793-0.795) | 0.020 (0.019-0.021) | 0.999 (0.999-0.999) | 279,849 | 72,584 | 323 | 1,505 |
| DeepSurv 35 | 2 | 0.870 (0.866-0.875) | 0.832 (0.820-0.843) | 0.777 (0.775-0.778) | 0.035 (0.035-0.037) | 0.998 (0.998-0.998) | 272,475 | 78,334 | 581 | 2,871 |
| DeepSurv 35 | 5 | 0.848 (0.845-0.851) | 0.817 (0.809-0.823) | 0.753 (0.752-0.754) | 0.071 (0.070-0.073) | 0.994 (0.994-0.995) | 260,637 | 85,598 | 1,470 | 6,556 |
| DeepSurv 35 | 10 | 0.810 (0.807-0.813) | 0.774 (0.767-0.779) | 0.727 (0.726-0.728) | 0.106 (0.104-0.108) | 0.987 (0.987-0.988) | 247,127 | 92,951 | 3,203 | 10,980 |
| DeepSurv 35 comp | 1 | 0.873 (0.866-0.881) | 0.847 (0.834-0.863) | 0.750 (0.748-0.751) | 0.017 (0.016-0.018) | 0.999 (0.999-0.999) | 264,249 | 88,184 | 279 | 1,549 |
| DeepSurv 35 comp | 2 | 0.871 (0.866-0.876) | 0.838 (0.826-0.850) | 0.759 (0.757-0.760) | 0.033 (0.032-0.034) | 0.998 (0.998-0.998) | 266,181 | 84,628 | 559 | 2,893 |
| DeepSurv 35 comp | 5 | 0.853 (0.850-0.857) | 0.823 (0.815-0.833) | 0.744 (0.743-0.746) | 0.069 (0.068-0.071) | 0.995 (0.994-0.995) | 257,685 | 88,550 | 1,418 | 6,608 |
| DeepSurv 35 comp | 10 | 0.818 (0.816-0.821) | 0.790 (0.786-0.799) | 0.715 (0.714-0.717) | 0.104 (0.103-0.106) | 0.988 (0.988-0.988) | 243,201 | 96,877 | 2,980 | 11,203 |
| XGBoost 2500 | 1 | 0.887 (0.881-0.894) | 0.833 (0.813-0.848) | 0.809 (0.808-0.810) | 0.022 (0.021-0.023) | 0.999 (0.999-0.999) | 285,014 | 67,421 | 305 | 1,523 |
| XGBoost 2500 | 2 | 0.877 (0.871-0.881) | 0.829 (0.816-0.843) | 0.800 (0.799-0.801) | 0.039 (0.038-0.041) | 0.998 (0.998-0.998) | 280,527 | 70,284 | 589 | 2,863 |
| XGBoost 2500 | 5 | 0.852 (0.849-0.856) | 0.827 (0.820-0.837) | 0.761 (0.760-0.763) | 0.074 (0.073-0.076) | 0.995 (0.995-0.995) | 263,466 | 82,771 | 1,385 | 6,641 |
| XGBoost 2500 | 10 | 0.811 (0.809-0.814) | 0.798 (0.792-0.805) | 0.716 (0.714-0.717) | 0.105 (0.103-0.107) | 0.988 (0.988-0.989) | 243,323 | 96,757 | 2,866 | 11,317 |
| XGBoost 35 | 1 | 0.880 (0.873-0.887) | 0.834 (0.818-0.851) | 0.791 (0.790-0.793) | 0.020 (0.019-0.021) | 0.999 (0.999-0.999) | 278,847 | 73,588 | 303 | 1,525 |
| XGBoost 35 | 2 | 0.870 (0.865-0.875) | 0.834 (0.822-0.844) | 0.778 (0.776-0.779) | 0.036 (0.034-0.037) | 0.998 (0.998-0.998) | 272,816 | 77,995 | 573 | 2,879 |
| XGBoost 35 | 5 | 0.845 (0.841-0.848) | 0.819 (0.810-0.828) | 0.749 (0.748-0.751) | 0.070 (0.069-0.072) | 0.994 (0.994-0.995) | 259,451 | 86,786 | 1,457 | 6,569 |
| XGBoost 35 | 10 | 0.805 (0.802-0.809) | 0.776 (0.769-0.783) | 0.721 (0.719-0.723) | 0.104 (0.102-0.106) | 0.987 (0.987-0.988) | 245,162 | 94,918 | 3,178 | 11,005 |
| Cox 400 | 1 | 0.874 (0.867-0.881) | 0.822 (0.804-0.839) | 0.784 (0.783-0.785) | 0.019 (0.019-0.020) | 0.999 (0.999-0.999) | 276,385 | 76,048 | 326 | 1,502 |
| Cox 400 | 2 | 0.869 (0.863-0.874) | 0.822 (0.809-0.835) | 0.778 (0.776-0.779) | 0.035 (0.034-0.036) | 0.998 (0.998-0.998) | 272,815 | 77,994 | 615 | 2,837 |
| Cox 400 | 5 | 0.848 (0.843-0.851) | 0.803 (0.795-0.811) | 0.768 (0.766-0.769) | 0.074 (0.072-0.076) | 0.994 (0.994-0.994) | 265,863 | 80,372 | 1,585 | 6,441 |
| Cox 400 | 10 | 0.810 (0.807-0.814) | 0.782 (0.776-0.789) | 0.718 (0.716-0.719) | 0.104 (0.102-0.105) | 0.988 (0.987-0.988) | 243,992 | 96,086 | 3,090 | 11,093 |
| Cox 35 | 1 | 0.858 (0.852-0.866) | 0.786 (0.769-0.805) | 0.793 (0.792-0.794) | 0.019 (0.018-0.020) | 0.999 (0.999-0.999) | 279,401 | 73,032 | 392 | 1,436 |
| Cox 35 | 2 | 0.855 (0.850-0.861) | 0.797 (0.784-0.810) | 0.777 (0.775-0.778) | 0.034 (0.033-0.035) | 0.997 (0.997-0.998) | 272,415 | 78,394 | 700 | 2,752 |
| Cox 35 | 5 | 0.837 (0.832-0.840) | 0.798 (0.787-0.807) | 0.743 (0.741-0.744) | 0.067 (0.066-0.069) | 0.994 (0.993-0.994) | 257,245 | 88,990 | 1,623 | 6,403 |
| Cox 35 | 10 | 0.800 (0.797-0.803) | 0.776 (0.769-0.782) | 0.705 (0.704-0.707) | 0.099 (0.097-0.100) | 0.987 (0.987-0.987) | 239,871 | 100,207 | 3,173 | 11,010 |
| FLS | 1 | 0.566 (0.559-0.576) | 0.156 (0.141-0.171) | 0.977 (0.976-0.977) | 0.033 (0.030-0.037) | 0.996 (0.995-0.996) | 344,145 | 8,288 | 1,543 | 285 |
| FLS | 2 | 0.549 (0.544-0.554) | 0.121 (0.109-0.131) | 0.977 (0.976-0.977) | 0.049 (0.044-0.053) | 0.991 (0.991-0.991) | 342,654 | 8,155 | 3,034 | 418 |
| FLS | 5 | 0.530 (0.527-0.533) | 0.083 (0.077-0.089) | 0.977 (0.977-0.978) | 0.078 (0.072-0.083) | 0.979 (0.978-0.979) | 338,326 | 7,909 | 7,362 | 664 |
| FLS | 10 | 0.521 (0.519-0.522) | 0.064 (0.060-0.068) | 0.978 (0.977-0.978) | 0.106 (0.099-0.111) | 0.962 (0.961-0.962) | 332,410 | 7,668 | 13,278 | 905 |
|  |  |  |  |  |  |  |  |  |  |  |

AUC=Area Under Curve, PPV=Positive Predictive Value, NPV= Negative Predictive Value, TN=True Negatives, FP=False Positives, FN=False Negatives, TP=True Positive
