# Supplementary material for: A clinical decision support tool for accurate hip fracture prediction: A nationwide cohort study
Source: PLoS Med. 2026 Aug 27;23(8):e1005190. doi: 10.1371/journal.pmed.1005190 (PMC13521376; doi:10.1371/journal.pmed.1005190)
Supplement: Supplementary file 13 [file pmed.1005190.s013.docx]

## S4 Table. Cohort characteristics for the top 35 variables – part 1

| **Variable** | **Variable Importance** | **All Patients** | **Incident Hip Fracture** | |  | |
| --- | --- | --- | --- | --- | --- | --- |
|  |  |  | **Yes** | **No** |  |  |
|  |  | **N=3,542,647** | **N=142,327** | **N=3,400,320** |  |  |
| Age | 205,227,144 | 65.9±11.0 | 77.5±9.9 | 65.4±10.8 |  |  |
| Female sex | 3,653,755 | 1,805,997 (51.0) | 91,458 (64.3) | 1,714,539 (50.4) |  |  |
| Prescriptions past yr | 3,290,822 |  |  |  |  |  |
| 0 |  | 687,655 (19.4) | 11,742 (8.3) | 675,913 (19.9) |  |  |
| 1-19 |  | 1,857,523 (52.4) | 58,574 (41.2) | 1,798,949 (52.9) |  |  |
| 20-73 |  | 834,716 (23.6) | 56,532 (39.7) | 778,184 (22.9) |  |  |
| 74 or more |  | 162,753 (4.6) | 15,479 (10.9) | 147,274 (4.3) |  |  |
| Marital status* | 3,083,375 |  |  |  |  |  |
| Unmarried |  | 535,448 (15.1) | 14,935 (10.5) | 520,513 (15.3) |  |  |
| Divorced |  | 621,262 (17.5) | 22,863 (16.1) | 598,399 (17.6) |  |  |
| Widow(er) |  | 417,372 (11.8) | 46,366 (32.6) | 371,006 (10.9) |  |  |
| Married |  | 1,858,377 (52.5) | 56,286 (39.5) | 1,802,091 (53.0) |  |  |
| Alcohol related disorder (F10) past 13 yrs | 2,664,679 | 79,844 (2.3) | 4,911 (3.5) | 74,933 (2.2) |  |  |
| Anti-dementia drug (N06D) past 5 yrs | 2,219,915 | 45,938 (1.3) | 6,463 (4.5) | 39,475 (1.2) |  |  |
| Hospital stays, total days past 2 yrs | 1,710,641 |  |  |  |  |  |
| 0 |  | 2,800,766 (79.1) | 92,848 (65.2) | 2,707,918 (79.6) |  |  |
| 1-4 |  | 301,844 (8.5) | 14,781 (10.4) | 287,063 (8.4) |  |  |
| 5-12 |  | 239,289 (6.8) | 16,839 (11.8) | 222,450 (6.5) |  |  |
| 13 or more |  | 200,748 (5.7) | 17,859 (12.5) | 182,889 (5.4) |  |  |
| Citizenship at birth | 1,503,817 |  |  |  |  |  |
| Sweden |  | 2,985,555 (84.3) | 128,212 (90.1) | 2,857,343 (84.0) |  |  |
| Nordic |  | 183,304 (5.2) | 7,145 (5.0) | 176,159 (5.2) |  |  |
| Europe |  | 204,704 (5.8) | 4,973 (3.5) | 199,731 (5.9) |  |  |
| Asia |  | 107,046 (3.0) | 1,199 (0.8) | 105,847 (3.1) |  |  |
| Other |  | 62,038 (1.8) | 798 (0.6) | 61,240 (1.8) |  |  |
| Prescriptions past yr, unique ATC-codes | 1,176,785 |  |  |  |  |  |
| 0 |  | 687,655 (19.4) | 11,742 (8.3) | 675,913 (19.9) |  |  |
| 1-6 |  | 1,728,257 (48.8) | 56,148 (39.4) | 1,672,109 (49.2) |  |  |
| 7-13 |  | 843,331 (23.8) | 53,184 (37.4) | 790,147 (23.2) |  |  |
| 14 or more |  | 283,404 (8.0) | 21,253 (14.9) | 262,151 (7.7) |  |  |
| Transplanted organ (Z094) past 10 yrs | 935,781 | 154,918 (4.4) | 11,802 (8.3) | 143,116 (4.2) |  |  |
| Fracture past yr | 889,273 | 85,845 (2.4) | 9,113 (6.4) | 76,732 (2.3) |  |  |
| Femoral neck fracture (S720) past yr | 783,141 | 7,811 (0.2) | 1,647 (1.2) | 6,164 (0.2) |  |  |
| Hip replacement (NFB49) past 13 yrs | 700,641 | 84,746 (2.4) | 4,424 (3.1) | 80,322 (2.4) |  |  |
| Sickness benefits* | -697,588 | 208,633 (5.9) | 2,929 (2.1) | 205,704 (6.0) |  |  |
| Outpatients visits past 5 yrs | 641,645 |  |  |  |  |  |
| 0 |  | 780,127 (22.0) | 18,191 (12.8) | 761,936 (22.4) |  |  |
| 1-3 |  | 1,032,358 (29.1) | 33,942 (23.8) | 998,416 (29.4) |  |  |
| 4-8 |  | 870,426 (24.6) | 39,722 (27.9) | 830,704 (24.4) |  |  |
| 9 or more |  | 859,736 (24.3) | 50,472 (35.5) | 809,264 (23.8) |  |  |

Cohort characteristics for the 35 most important variables in DeepSurv prediction model. Continuous variables presented with mean ± SD and categoric variables with n (%). Variable importance represents change in AUC ( x 1,000,000,000) when randomly permutated, i.e. how much AUC would have changed if the variable had no predictive value. *=missing values treated as separate categories (marital status 3.1%, sickness benefits 3.1% and education level 4.2%)

## S3 Table. Cohort characteristics for the top 35 variables – part 2

|  |  | **All patients** | **Incident hip fracture** | |  |
| --- | --- | --- | --- | --- | --- |
|  | **Variable** |  | **Yes** | **No** |  |
| **Variable** | **Importance** | **N=3,542,647** | **N=142,327** | **N=3,400,320** |  |
| Education level* | 480,868 |  |  |  |  |
| Elementary school incomplete |  | 660,938 (18.7) | 53,689 (37.7) | 607,249 (17.9) |  |
| Elementary school complete |  | 352,880 (10.0) | 11,528 (8.1) | 341,352 (10.0) |  |
| Upper Secondary / High School |  | 1,446,848 (40.8) | 50,110 (35.2) | 1,396,738 (41.1) |  |
| University / College less than 3 yrs |  | 131,782 (3.7) | 2,019 (1.4) | 129,763 (3.8) |  |
| University / College more than 3 yrs |  | 761,932 (21.5) | 20,107 (14.1) | 741,825 (21.8) |  |
| PhD |  | 37,987 (1.1) | 845 (0.6) | 37,142 (1.1) |  |
| Pain in throat and chest (R07) past 10 yrs | 368,214 | 360,230 (10.2) | 17,012 (12.0) | 343,218 (10.1) |  |
| Parkinson disease (G209) past 10 yrs | 217,183 | 16,997 (0.5) | 2,229 (1.6) | 14,768 (0.4) |  |
| Cataract past 13 yrs | 191,075 | 395,126 (11.2) | 39,995 (28.1) | 355,131 (10.4) |  |
| Charlson comorbidity index | 172,541 |  |  |  |  |
| 0 |  | 2,377,896 (67.1) | 66,366 (46.6) | 2,311,530 (68.0) |  |
| 1 |  | 416,840 (11.8) | 24,822 (17.4) | 392,018 (11.5) |  |
| 2 |  | 426,323 (12.0) | 25,563 (18.0) | 400,760 (11.8) |  |
| 3 |  | 153,047 (4.3) | 12,361 (8.7) | 140,686 (4.1) |  |
| 4 or more |  | 168,541 (4.8) | 13,215 (9.3) | 155,326 (4.6) |  |
| Atrial fibrillation/flutter (I489) past 13 yrs | -166,637 | 228,415 (6.4) | 18,468 (13.0) | 209,947 (6.2) |  |
| Anti-Parkinson drugs (N04BA) past yr | 154,084 | 25,470 (0.7) | 2,975 (2.1) | 22,495 (0.7) |  |
| Femoral fracture surgery (NFJ59) past 2 yrs | 120,802 | 4,942 (0.1) | 879 (0.6) | 4,063 (0.1) |  |
| Iron preparations (B03A) past 5 yrs | 78,500 | 181,974 (5.1) | 13,766 (9.7) | 168,208 (4.9) |  |
| Chronic kidney disease (N189) past 10 yrs | -68,800 | 28,424 (0.8) | 2,381 (1.7) | 26,043 (0.8) |  |
| Hypotension (I95) past 10 yrs | 48,200 | 28,838 (0.8) | 2,992 (2.1) | 25,846 (0.8) |  |
| Hip contusion (S700) past 2 yrs | 37,400 | 12,505 (0.4) | 1,533 (1.1) | 10,972 (0.3) |  |
| Blood transfusion (DR029) past 10 yrs | 37,000 | 74,386 (2.1) | 6,301 (4.4) | 68,085 (2.0) |  |
| Penicillin (J01C) past 5 yrs | 30,600 | 1,645,860 (46.5) | 73,453 (51.6) | 1,572,407 (46.2) |  |
| GnRH analogues (L02AE) past 2 yrs | -15,900 | 18,508 (0.5) | 1,748 (1.2) | 16,760 (0.5) |  |
| Metastasis fr bone/marrow (C795) past 10 yrs | -10,400 | 6,650 (0.2) | 317 (0.2) | 6,333 (0.2) |  |
| Morphine (N02AA01) past yr | 8,890 | 43,055 (1.2) | 3,512 (2.5) | 39,543 (1.2) |  |
| Osmotically acting laxatives (A06AD) past yr | -786 | 223,763 (6.3) | 18,004 (12.6) | 205,759 (6.1) |  |
| Atherosclerosis (I70) past 10 yrs | -359 | 39,778 (1.1) | 3,469 (2.4) | 36,309 (1.1) |  |
| Clomethiazole (N05CM02) past 2 yrs | -160 | 12,930 (0.4) | 1,328 (0.9) | 11,602 (0.3) |  |

Cohort characteristics for the 35 most important variables in DeepSurv prediction model. Continuous variables presented with mean ± SD and categoric variables with n (%). Variable importance represents change in AUC
( x 1,000,000,000) when randomly permutated, i.e. how much AUC would have changed if the variable had no predictive value. *=missing values treated as separate categories (marital status 3.1%, sickness benefits 3.1% and education level 4.2%)
