# Supplementary material for: A clinical decision support tool for accurate hip fracture prediction: A nationwide cohort study
Source: PLoS Med. 2026 Aug 27;23(8):e1005190. doi: 10.1371/journal.pmed.1005190 (PMC13521376; doi:10.1371/journal.pmed.1005190)
Supplement: Supplementary file 14 [file pmed.1005190.s014.docx]

# S5 Table. Values from precision-recall curves for 2, 5 and 10 years prediction

Performance metrics in hold-out cohort (N=354,261) for hip fracture prediction at 2, 5 and 10 years for Cox 35, Cox 400, DeepSurv 35 and DeepSurv 2500 at 80% recall (sensitivity) and maximum precision (positive predictive value). TP=True Positives. FP=False Positives. TN=True Negatives. FN=False Negatives.

| **Point in curve** | **Follow-up** | **Model** | **Threshold** | **TP** | **FP** | **TN** | **FN** | **Sensitivity** | **Specificity** | **Precision** |
| --- | --- | --- | --- | --- | --- | --- | --- | --- | --- | --- |
| 80% sensitivity | 2 years | DeepSurv 35 | 1,3% | 2,759 | 66,754 | 284,054 | 694 | 79,9% | 81,0% | 4,0% |
| 80% sensitivity | 2 years | DeepSurv 2500 | 1,5% | 2,779 | 62,139 | 288,669 | 674 | 80,5% | 82,3% | 4,3% |
| 80% sensitivity | 2 years | Cox 35 | 1,3% | 2,771 | 81,096 | 269,712 | 682 | 80,2% | 76,9% | 3,3% |
| 80% sensitivity | 2 years | Cox 400 | 1,3% | 2,761 | 69,523 | 281,285 | 692 | 80,0% | 80,2% | 3,8% |
| Max precision | 2 years | DeepSurv 35 | 3,8% | 1,075 | 14,008 | 336,800 | 2,378 | 31,1% | 96,0% | 7,1% |
| Max precision | 2 years | DeepSurv 2500 | 5,2% | 915 | 11,754 | 339,054 | 2,538 | 26,5% | 96,6% | 7,2% |
| Max precision | 2 years | Cox 35 | 6,9% | 402 | 5,188 | 345,620 | 3,051 | 11,6% | 98,5% | 7,2% |
| Max precision | 2 years | Cox 400 | 5,2% | 1,045 | 13,342 | 337,466 | 2,408 | 30,3% | 96,2% | 7,3% |
| 80% sensitivity | 5 years | DeepSurv 35 | 2,5% | 6,448 | 80,369 | 265,858 | 1,586 | 80,3% | 76,8% | 7,4% |
| 80% sensitivity | 5 years | DeepSurv 2500 | 2,7% | 6,421 | 74,997 | 271,230 | 1,613 | 79,9% | 78,3% | 7,9% |
| 80% sensitivity | 5 years | Cox 35 | 2,9% | 6,426 | 89,811 | 256,416 | 1,608 | 80,0% | 74,1% | 6,7% |
| 80% sensitivity | 5 years | Cox 400 | 3,0% | 6,437 | 79,979 | 266,248 | 1,597 | 80,1% | 76,9% | 7,4% |
| Max precision | 5 years | DeepSurv 35 | 6,4% | 3,096 | 23,444 | 322,783 | 4,938 | 38,5% | 93,2% | 11,7% |
| Max precision | 5 years | DeepSurv 2500 | 8,1% | 2,959 | 22,293 | 323,934 | 5,075 | 36,8% | 93,6% | 11,7% |
| Max precision | 5 years | Cox 35 | 9,9% | 2,011 | 14,293 | 331,934 | 6,023 | 25,0% | 95,9% | 12,3% |
| Max precision | 5 years | Cox 400 | 9,3% | 2,783 | 19,915 | 326,312 | 5,251 | 34,6% | 94,2% | 12,3% |
| 80% sensitivity | 10 years | DeepSurv 35 | 3,5% | 11,388 | 102,614 | 237,464 | 2,795 | 80,3% | 69,8% | 10,0% |
| 80% sensitivity | 10 years | DeepSurv 2500 | 3,6% | 11,334 | 96,508 | 243,570 | 2,849 | 79,9% | 71,6% | 10,5% |
| 80% sensitivity | 10 years | Cox 35 | 5,0% | 11,369 | 110,164 | 229,914 | 2,814 | 80,2% | 67,6% | 9,4% |
| 80% sensitivity | 10 years | Cox 400 | 5,3% | 11,366 | 102,621 | 237,457 | 2,817 | 80,1% | 69,8% | 10,0% |
| Max precision | 10 years | DeepSurv 35 | 9,0% | 5,471 | 32,648 | 307,430 | 8,712 | 38,6% | 90,4% | 14,4% |
| Max precision | 10 years | DeepSurv 2500 | 10,3% | 4,869 | 28,567 | 311,511 | 9,314 | 34,3% | 91,6% | 14,6% |
| Max precision | 10 years | Cox 35 | 12,3% | 3,687 | 21,203 | 318,875 | 10,496 | 26,0% | 93,8% | 14,8% |
| Max precision | 10 years | Cox 400 | 12,6% | 4,356 | 24,822 | 315,256 | 9,827 | 30,7% | 92,7% | 14,9% |
